# Fold-recognition and comparative modeling of human α2,3-sialyltransferases reveal their sequence and structural similarities to CstII from *Campylobacter jejuni*

**DOI:** 10.1186/1472-6807-6-9

**Published:** 2006-04-19

**Authors:** MS Sujatha, Petety V Balaji

**Affiliations:** 1 School of Biosciences and Bioengineering, Indian Institute of Technology Bombay, Powai, Mumbai 400 076, India

## Abstract

**Background:**

The 3-D structure of none of the eukaryotic sialyltransferases (SiaTs) has been determined so far. Sequence alignment algorithms such as BLAST and PSI-BLAST could not detect a homolog of these enzymes from the protein databank. SiaTs, thus, belong to the hard/medium target category in the CASP experiments. The objective of the current work is to model the 3-D structures of human SiaTs which transfer the sialic acid in α2,3-linkage viz., ST3Gal I, II, III, IV, V, and VI, using fold-recognition and comparative modeling methods. The pair-wise sequence similarity among these six enzymes ranges from 41 to 63%.

**Results:**

Unlike the sequence similarity servers, fold-recognition servers identified CstII, a α2,3/8 dual-activity SiaT from *Campylobacter jejuni *as the homolog of all the six ST3Gals; the level of sequence similarity between CstII and ST3Gals is only 15–20% and the similarity is restricted to well-characterized motif regions of ST3Gals. Deriving template-target sequence alignments for the entire ST3Gal sequence was not straightforward: the fold-recognition servers could not find a template for the region preceding the L-motif and that between the L- and S-motifs. Multiple structural templates were identified to model these regions and template identification-modeling-evaluation had to be performed iteratively to choose the most appropriate templates. The modeled structures have acceptable stereochemical properties and are also able to provide qualitative rationalizations for some of the site-directed mutagenesis results reported in literature. Apart from the predicted models, an unexpected but valuable finding from this study is the sequential and structural relatedness of family GT42 and family GT29 SiaTs.

**Conclusion:**

The modeled 3-D structures can be used for docking and other modeling studies and for the rational identification of residues to be mutated to impart desired properties such as altered stability, substrate specificity, etc. Several studies in literature have focused on the development of tools and/or servers for the large-scale/automated modeling of 3-D structures of proteins. In contrast, the present study focuses on modeling the 3-D structure of a specific protein of interest to a biochemist and illustrates the associated difficulties. It is also able to establish a sequence/structure relationship between sialyltransferases of two distinct families.

## Background

Sialyltransferases (SiaTs) catalyze the transfer of sialic acid from CMP-Neu5Ac donor substrate to the terminal non-reducing saccharide of glycoproteins or glycolipids [1-4]. They are type II transmembrane proteins with a short, cytoplasmic N-terminal domain followed by a transmembrane domain, a flexible stem region of variable length and a catalytic domain. SiaTs use a variety of glycoconjugates as acceptor substrates in vivo; they can also use mono-, di- or oligo-saccharides as acceptor substrates in vitro. The sialic acid residue can be transferred in α2,3-linkage to Gal, in α2,6-linkage to Gal, GlcNAc or GalNAc and in α2,8-/α2,9-linkage to another sialic acid. SiaTs constitute a superfamily and have been further classified as ST3 (α2,3), ST6Gal (α2,6 to Gal), ST6GalNAc (α2,6 to GalNAc) and ST8 (α2,8/9) families on the basis of the linkage in which sialic acid is transferred [5]. Further classification viz., ST3Gal I, ST3Gal II, etc., is based on acceptor specificity and amino acid sequence.

Eukaryotic SiaTs share four sequence motifs in their catalytic domain; these are L- (large), S- (small), and VS- (very small) motifs [6] and motif III [7]. The roles of conserved residues found within the sialylmotifs have been investigated by site-specific mutation analyses in ST6Gal I. Residues in the L-motif have been implicated in binding donor substrate [8] whereas those in the S-motif have been implicated in binding both the donor and acceptor substrates [8,9]. Mutation of the conserved His residue in the VS-motif to Lys led to loss of activity [10]. Mutating the conserved histidine in VS-motif to alanine gave rise to an enzyme with no activity. Similarly, mutations of histidine and tyrosine residues in motif III to alanine in ST3Gal I also resulted in complete loss of enzyme activity [7]. These motifs are common to all SiaTs and are thus expected to be involved in shared functions such as donor substrate binding, folding and maintaining proper 3-D structure, and catalysis.

The residues that are not conserved across the families are expected to generate differential acceptor specificity, oligomerization, protein-protein interaction, etc. A recent sequence analysis study identified linkage- (family-) specific sequence motifs [11]. Two motifs were found to be unique to the ST3Gal family: ^185^TTx(4)YPE^193 ^and ^209^FKxxDxxW^216 ^(human ST3Gal I numbering; accession no. AAA36612). The former motif is contiguous to the L-motif. These motifs, being specific to the ST3 family, are expected to contribute to the characteristic linkage- and acceptor substrate-specificities of the family members [11].

Knowledge of the 3-D structure of SiaTs is crucial to understand the origin of the substrate specificity and to rationalize the site-specific mutation data on the conserved residues in sialylmotifs. This knowledge will also help in establishing the structure-function relationship in this family of proteins and thereby in generating SiaTs with modified substrate specificity for chemo-enzymatic synthesis of oligosaccharides. However, the 3-D structure of none of the eukaryotic SiaTs is known to date. In view of this, the 3-D structures of six human SiaTs belonging to ST3Gal family have been modeled using fold-recognition and comparative modeling methods. Six different ST3Gals were considered for modeling since their pair-wise sequence similarity ranges from 41 to 66% and they are expected to share the same fold because of their biochemical functional similarities.

Fold-recognition servers identified CstII, a α2,3/8 dual-activity SiaT from *Campylobacter jejuni *as the homolog of all the six ST3Gals. The generated 3-D models have acceptable stereochemistry. It was also possible to provide a structure-based rationalization for the functional behavior of many of the site-specific mutants. Independent modeling of the six ST3Gals leading to the similar structures enhanced the confidence levels in the generated models. The results also establish that the GT29 and GT42 family SiaTs share sequence and structural similarities.

## Results and discussion

### Sequence similarity between ST3Gals

**Figure 1:**
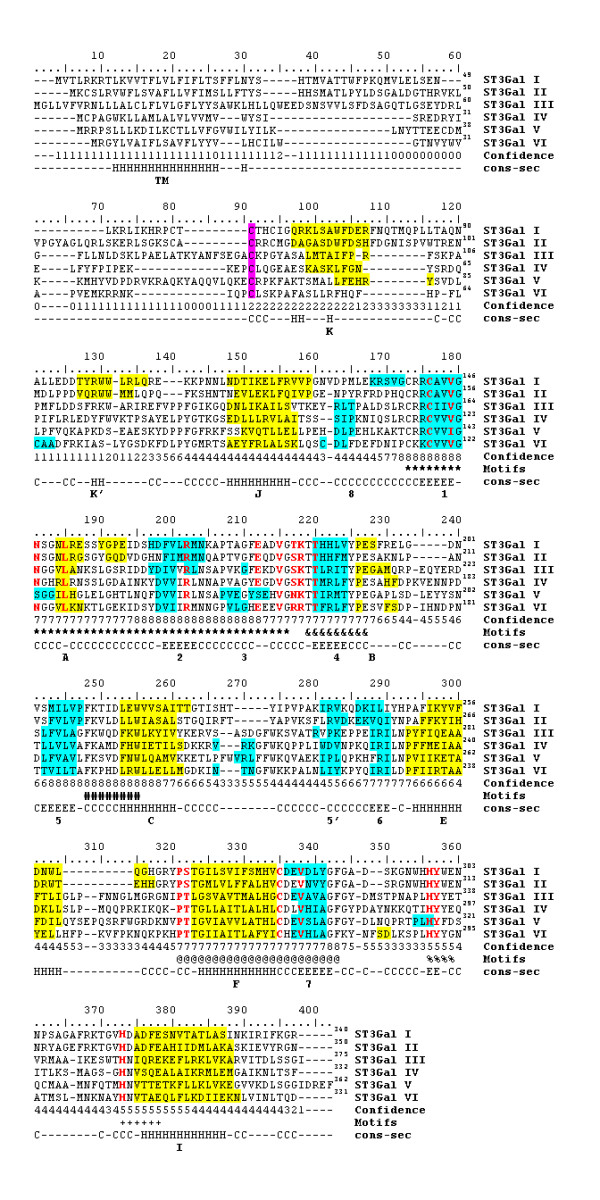
Multiple sequence alignment of ST3Gals (Table 1) obtained from the Tcoffee server [41]. A hyphen (" - ") indicates a one-residue gap. The ruler at the top is for the entire alignment and has no reference to any individual sequence; the latter are numbered on the right. The residues constituting the various motifs are marked below: L-motif (alignment position 173–216, denoted by *), linkage-specific motifs (219–227, denoted by & and 248–255, denoted by #), S-motif (321–343, denoted by @), motif III (356–359, denoted by %) and VS-motif (373–378, denoted by +). The structural and functionally important residues identified by mutation studies (Table 2) have been highlighted in red with bold font. *Confidence *is the confidence score given by Tcoffee. The regions having the same secondary structure in all the ST3Gals are also shown (marked *cons-sec *for consensus secondary structure). The names of the various strands and helices, indicated below the consensus secondary structure, are the same as those of the corresponding regions in CstII [13]. Residues highlighted in yellow and cyan constitute α-helices and β-strands, respectively, (as identified by SwissPDBviewer/RasMol) in at least one of the top models. The conserved cysteine residue in the stem region is highlighted in pink.

Pairwise sequence similarity between ST3Gal I, II, III, IV, V and VI ranges from 41 to 66% (see Additional file 1). The similarity is higher (45–80%) in the region from the L-motif up to the C-terminus. ST3Gal I and II are more similar to each other than they are to other four ST3s as has been noted previously [5]. The six ST3Gal sequences were also multiply aligned using the TCoffee server (Figure 1). The level of confidence in the alignment is quite high, as judged by the confidence scores generated by the TCoffee algorithm, except in regions encompassing the stem, transmembrane and N-terminal cytoplasmic domains (Figure 1). The motifs of the SiaT superfamily (L-, S- and VS-motifs and motif III) and linkage-specific motifs of the ST3 family align with each other. A cysteine residue in the stem region is conserved in all the six ST3Gals (Figure 1).

### Secondary structure prediction

A consensus secondary structure was derived for each SiaT based on the results from eight secondary structure prediction servers (see Additional file 2). The region predicted by the TMHMM server as the transmembrane domain is predicted to be helical in all the ST3Gals. The sequence and length of the region between the transmembrane domain and L-motif in the six ST3Gals are different; this region has only helices but the number of helices varies between 3 and 5. The significance of this variability and its relevance (if any) to differences in acceptor substrate specificity are as yet unknown. The order of occurrence of the secondary structural elements from the L-motif onwards is very nearly the same in all the ST3Gals. The L-motif region is made of coils and strands. The S-motif begins with a helix, immediately followed by a strand. The six-residue-long VS-motif is partly helical. The region between the L- and S-motifs has a mixture of strands and helices. Of the two ST3 family-specific motifs, TTx(4)YPE is part of a strand and FKxxDxxW is in coil conformation.

Overall, 25–32% of residues are in helices and 9–12% residues in strands. The conservation of the nature and order of occurrence of secondary structural elements is strongly suggestive of the conservation of the overall fold in these ST3Gals. It can be inferred from the predicted secondary structures that ST3Gals belong to the α/β class, as defined in the SCOP database [12]. Other glycosyltransferases (GlyTs) whose 3-D structures have been determined so far also belong to the same class. Within this class, there are three fold types designated as nucleotide-diphospho-sugar transferases, UDP-glycosyltransferase/glycogen phosphorylase and α-2,3/8-sialyltransferase CstII.

### Template identification by fold-recognition servers

Two approaches were employed to identify the potential templates: (1) Submitting a multiple sequence alignment (MSA) of all the six ST3Gals and (2) Submitting each of the six ST3Gal sequences individually. In the former, MSA for the entire sequence from N- to C-terminus (Figure 1) was submitted to the FUGUE server; the templates that were identified had very low confidence levels (Z-score for the top hit = 2.54; *guess*). Even the GeneSilico metaserver identifies templates with very low confidence levels (pcons5 score for the top hit = 0.15; *unreliable*); the α2,3/8 dual-activity sialyltransferase CstII from *Campylobacter jejuni *(PDB id 1RO7 [13]; referred to as CstII henceforth) has a pcons5 score of 0.09. However, the alignment with CstII began from only the L-motif onwards of ST3Gals; no template was identified for the region preceding the L-motifs, most likely due to the very low sequence similarity in this region of the ST3Gals. In view of these, MSA starting from the L-motif onwards up to the C-terminus was submitted to these servers. Both the servers identify CstII as the top hit (Z-score = 5.2; *likely *and pcons5 score = 0.32; *unreliable*).

**Figure 2:**
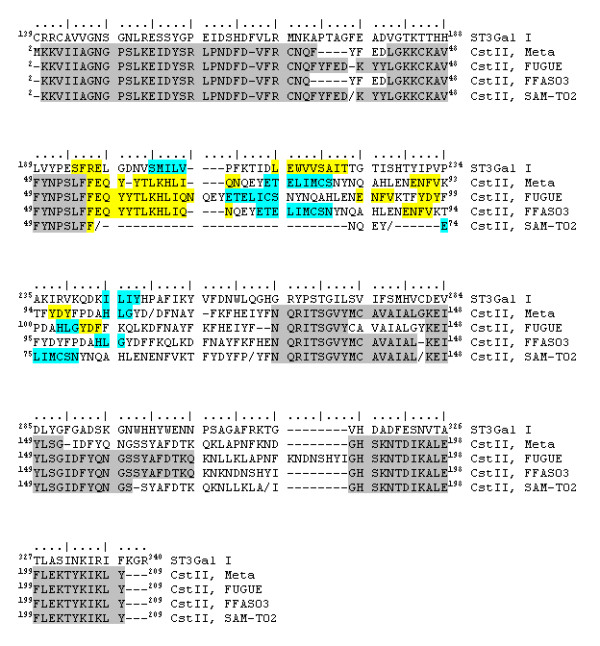
Target (ST3Gal I) – template (CstII, PDB id 1RO7) sequence alignment generated by the fold-recognition servers. The alignments were generated by the indicated servers when ST3Gal I sequence from the L-motif region till the C-terminus was used as query. Regions of alignment that are same for the four servers are highlighted. The alignments for the region spanning residues 215 to 254 of ST3Gal I generated by the four servers are different. The mismatch in alignment of secondary structures of target -- template are also seen in these regions. The helices are highlighted as yellow and the strands are shown in blue (as in Figure 1). Such a disagreement for this region is seen in the case of other ST3Gals also.

In the second approach, complete sequence from N- to C-terminus of the six ST3Gals was used separately as query to search for homologs in the PDB database using BLAST and PSI-BLAST. No significant hits were obtained. Among the fold-recognition servers, only FFAS03 and the GeneSilico metaserver identified CstII as a hit and the alignment began from the L-motif region of ST3Gals. However, if only the sequence from L-motif onwards is used as query, then even FUGUE and SAM-T02 servers identify CstII as the possible template with a high level of confidence (see Additional file 3). The template-target alignments generated for motif regions (Figure 1) when ST3Gal sequences were submitted individually were same as that obtained by submitting the multiple sequence alignment. In all the cases, the secondary structures of the target and template residues in the alignment regions 250–290 (Figure 1) were entirely different (Figure 2).

The alignments generated by different servers do not agree with each other in some regions. The disagreement was resolved based on secondary structure states of the residues at some regions. For example, the residues 215–254 of ST3Gal I are aligned differently with CstII by the four fold-recognition servers (Figure 2); even the secondary structure states of the aligned residues are different (Figure 2). A similar mismatch was found for the corresponding region of other ST3Gals also. For such regions, other template(s) that would satisfy the predicted secondary structure in that region were identified by submitting only the relevant part of the sequence to the fold-recognition servers and/or PSI-BLAST (see Additional file 4). Thus, the use of pair-wise target-template alignment seems to be more appropriate than deriving templates based on multiple sequence alignment [14].

### Sequence alignment for regions preceding L-motif in ST3Gals

The membrane-association region in CstII is at the C-terminus [13] unlike the human SiaTs, which have the transmembrane domain at the N-terminus (see Additional file 5). Consequently, the N-terminus of ST3Gals (~150 residues containing the cytoplasmic and transmembrane domains and the stem region) and the C-terminus of CstII (~90 residues; containing the membrane-association region) are left out of alignment generated by the fold-recognition servers. The alignment begins with the N-terminus of CstII and L-motif of ST3Gals; specifically, Lys2 of CstII aligns with Arg140 (ST3Gal I numbering), the second residue of the L-motif (Figure 2). Reversing the directionality of the polypeptide chain in the C-terminus of CstII (i.e., from residue 210 onwards) sets the transmembrane domain of ST3Gal in a position equivalent to the membrane-association region of CstII. The N-terminal region preceding the L-motif of ST3Gals was thus modeled following the Cα-trace of the CstII C-terminus in reverse direction. A considerable amount of similarity in secondary structures was also observed in these regions.

### Modeling 3-D structures starting from alignments

The 3-D structure of CstII (PDB id 1RO7; A chain) is the main template for modeling the 3-D structures of all the ST3Gals. Additional templates have been used for regions, which do not have a match in CstII by separately submitting the sequence of these regions to fold-recognition servers and PSI-BLAST (see Additional file 6). Even after this step, suitable templates could not be found for some regions immediately following the transmembrane domain; these regions were not modeled (Table 1). The combined sequence alignments (see Additional file 6) were used to model the 3-D structures of ST3Gals. Only the backbone conformation of the template is taken, and side chains are modeled independently, in regions where the template – target sequences disagree. Modeller uses a loop algorithm to model regions for which no template is specified. Twenty-five models were generated for each ST3Gals. The different structures vary in their backbone conformation, especially in regions that did not have a template, and in side chain conformations.

### Stereochemical evaluation of the predicted models

The stereochemical properties and quality of all the models were evaluated by MODELLER, PROCHECK and Verify3D (see Additional file 7). Three to four models were selected for each ST3Gal based on these evaluations. For all the selected models, the value of the objective function, reported as current energy by MODELLER, is in the same range as that if the template is aligned with its own sequence. On an average, 87% of the residues are found in the allowed region of Ramachandran map; PROCHECK considers the model to be very good if it has 90% of the residues in the most favored region. The inter-atomic distances are within acceptable range. Verify3D score is greater than zero for the region from the L-motif onwards but the score drops below 0 for certain regions preceding the L-motif. The models were also evaluated using Colorado3D server, which facilitates the change of amino acid window size when calculating the overall score. Two window sizes, 5 and 21, were used to calculate the average Verify3D and ProsaII score per residue for each of the top models and 25 models generated for the template. The scores calculated using these two window sizes were found to be very similar (see Additional file 7). The template and target models were rendered with the residues color-coded based on ProsaII (see Additional file 8) and verify3D (see Additional file 9) scores. With ProsaII score-based coloring, most of the residues are green and yellow (i.e., average score) in both the target and template proteins (see Additional file 8). With verify3D score-based coloring, even the template proteins has residues in red color (i.e., bad score) although the number of such residues are more in the targets (see Additional file 9).

### Characterization and comparison of modeled ST3Gal structures

**Figure 3:**
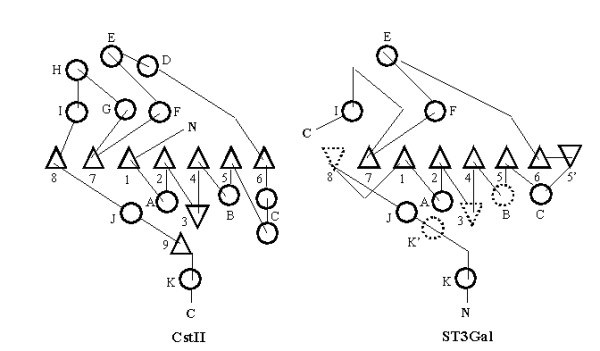
Topology diagrams for CstII (PDB ID: 1RO7) and modeled ST3Gals. Triangles and circles represent strands and helices, respectively. Strands 5' and 8 are antiparallel to other strands in ST3Gals and are hence inverted. Certain helices and strands are not found in all the modeled ST3Gals (Figure 1) and these are represented by dotted lines. The nomenclature used to identify strands and helices in CstII are the same as those used by Chiu et al. [13]. The equivalent secondary structure elements in ST3Gals are identified by the same names. Strand 9 and helices D, G and H are absent in ST3Gals; strand 5' and helix K' are absent in CstII. Note that the region of the polypeptide from strand 8 till helix K is at the C-terminus in CstII but they are at the N-terminus in ST3Gals; hence, the direction of the polypeptide chain is opposite to one another in this region.

**Figure 4:**
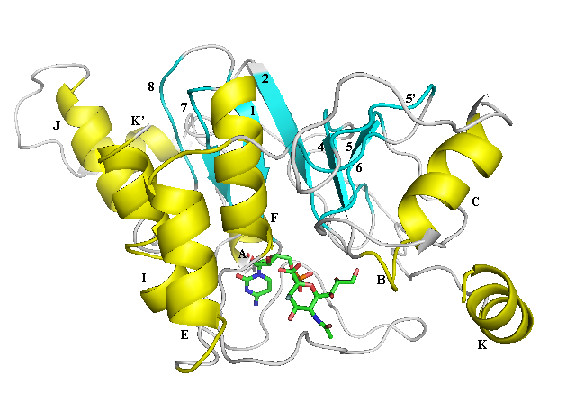
Cartoon diagram of human ST3Gal I modeled using the structure of CstII (PDB id 1RO7). Helices (A, B, C, E, F, I, J, K and K'; in yellow) and strands (β1, β2, β4, β5, β5', β6, β7 and β8; in cyan) have been given the same names as those of corresponding helices and strands in CstII. Residues constituting these helices and strands are given in Figure 1. The location of CMP-3-fluoro-NeuNAc (stick representation; carbon, green; oxygen, red; nitrogen, blue) has been derived by superposition of the modeled structure on that of the CstII- CMP-3-fluoro-NeuNAc complex. The structure was rendered using PyMol.

The ST3Gal fold is characterized by a six-stranded (β7, β1, β2, β4, β5 and β6; Figure 3) parallel β-sheet flanked on the two sides by strands β8 and β5' in an antiparallel orientation; strand β8 is present in only some ST3Gals (Figure 1). Helices E, F and I share a common interface and are in spatial proximity of strands β1, β2, β4 and β5 (Figure 4). Helices A and B are very small i.e., 3 to 4 residue long. Helices B and K' are found in only some ST3Gals.

**Figure 5:**
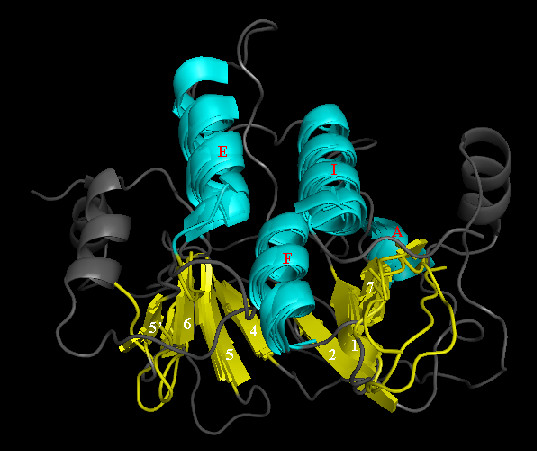
3-D rendering of the backbone traces of ST3Gal I, II, III, IV, V and VI superposed on each other using SwissPDBViewer. Helices A, E, F and I (all in cyan) and strands β1, β2, β4, β5, β5', β6 and β7 (all in yellow) superpose well on each other. Residues constituting these helices and strands are given in Figure 1. Loops and other helices/strands have different conformations in the six proteins and hence have been shown for only ST3Gal I (dark gray). PyMol was used for rendering.

The 3-D structures of ST3Gals compare well with each other to a large extent. Strands β7, β1, β2, β4, β5 andβ6 and helices E, F and I in various ST3Gals superpose well on each other (Figure 5). The length of the loop region between helices E and F is variable (Figure 1): it is shorter in ST3Gal I and II compared to that in the other four ST3Gals. It has been reported that ST3Gal I and II do not bind substrates that contain GlcNAc attached to terminal galactose whereas the other four do bind such substrates, albeit with varying affinities [15-20]. The relationship between the size of H6-H7 loop and the observed differences in the acceptor substrate specificities needs experimental validation. The conformation of the region from helix C to strand β6 also varies in different ST3Gals. This difference is due to differences in the amino acid sequences, which, in turn, required the use of different templates for modeling these regions.

### Comparison of the modeled structures with CstII structure

The modeled 3-D structures of ST3Gals are similar to, but not exactly same as, that of CstII (Figure 3). The similarity is to be expected since CstII was the main template for deriving the models. Helix B is 8–10 residues long in CstII; in ST3Gals, it is only a helical loop formed by a few residues in the alignment region 226–231 (Figure 1). Helix J is not as prominent in CstII as it is in the modeled ST3Gals. The average RMS deviation between the target (ST3Gals) and template (CstII) structures is calculated to be 1.9 Å by the SSM server and 2.4 Å by the DALI server (see Additional file 10). The 3-D structure of no other protein was found to be similar to that of ST3Gals by the SSM and DALI servers.

### Residues involved in binding to CMP-Neu5Ac, the donor substrate

**Figure 6:**
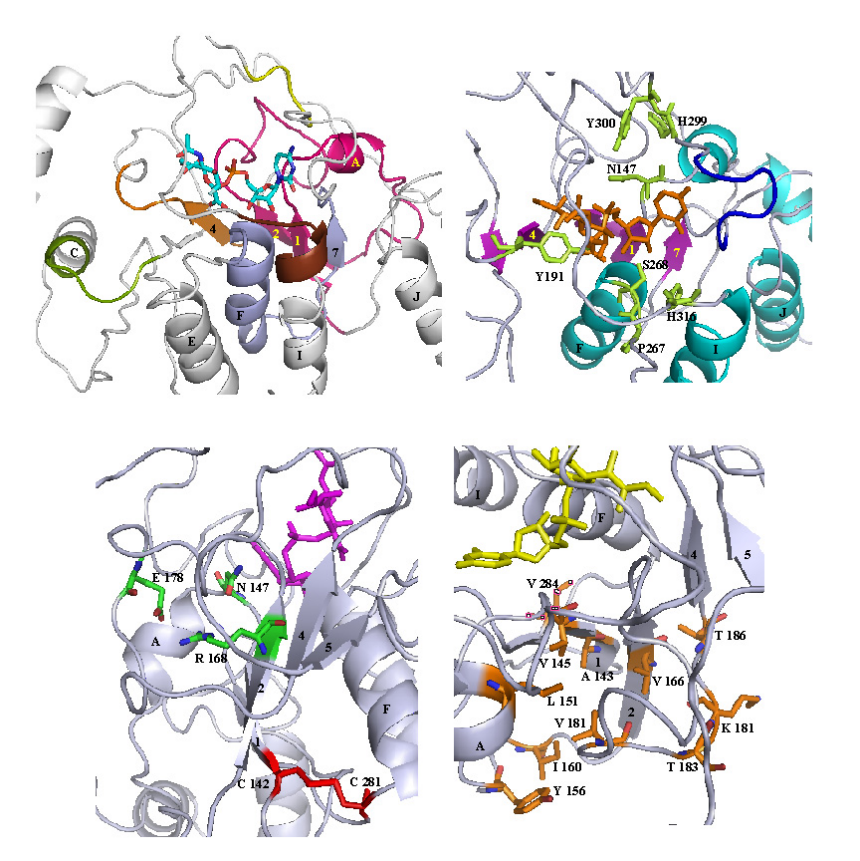
3-D renderings of ST3Gal I model highlighting the various functionally and structurally important residues and regions. The location of the ligand CMP-3-fluoro-NeuNAc as shown in these renderings has been derived by superposition of the modeled structure on that of the CstII- CMP-3-fluoro-NeuNAc complex. (**A, top left**) 3-D rendering showing the L-motif (magenta), S-motif (purple), VS-motif (burgundy), motif III (yellow), linkage-specific motifs TTx(4)YPE (orange) and FKxxDxxW (green) and (stick diagram). (**B, top right**) Location of some of the amino acid residues (colored green) whose roles have been investigated by site-directed mutagenesis studies (Table 2). The loop proposed in this study as important for nucleotide binding is shown in blue. The nomenclature used for identifying helices (cyan) and strands (magenta) is as that in Figure 1. (**C, bottom left**) The interactions of the conserved Arg168 with Asn147 and Glu178. All three residues are buried within the protein. The disulphide bridge (colored red) proposed as required for structural stability has also been displayed. The ligand is in pink. (**D, bottom right**) 3-D rendering showing the cluster of apolar residues proposed as important for maintaining the 3-D structure (Table 2). Note that not all these residues are in direct contact with the ligand.

CstII and ST3Gals are both sialyltransferases and use the same donor substrate, CMP-Neu5Ac. The crystal structure of CstII has been determined in complex with the donor substrate analog, CMP-3-fluoro-NeuNAc (PDB id 1RO7) [13]. The modeled ST3Gal structures were superposed on the structure of CstII; for this purpose, the backbone atoms of the residues constituting the L-, S- and VS-motifs were used as reference atoms. This enabled the identification of residues that are likely to interact with CMP-Neu5Ac in ST3Gals. The residues that are found within 5 Å from CMP-Neu5Ac were found to be part of the L-, S- and VS-motifs, motif III and one of the ST3Gal family-specific motifs viz., TTx(4)YPE (Figure 6A). The second family-specific motif FKxxDxxW is in spatial proximity of TTx(4)YPE and seems to have a role in binding the acceptor substrate (Figure 6A). In this putative binding mode, the loop between β7 and helix I is near cytosine, beginning of L-motif is near ribose, Tyr300 (ST3Gal I numbering) is close to phosphate, middle of L-motif is close to phosphate and sialic acid, and Tyr191 (ST3Gal I numbering), beginning of S-motif, His of VS-motif are close to sialic acid (Figure 6B).

### Location of residues whose functional importance has been studied by site-specific mutations

Site-directed mutagenesis has been used to investigate the role of several residues conserved in SiaT superfamily [7-10,21]. Quantitative analysis of rat ST6Gal I indicated the presence of only one disulphide bond although the enzyme has seven cysteine residues [21]. All the modeled ST3Gals have one disulphide bond between two conserved cysteine residues, one present at the beginning of the L-motif and the other in the middle of the S-motif (Figure 6C). These two cysteine residues come in spatial proximity of each other when no specific constraints were used for the purpose of bringing them together. This disulfide bridge holds the β-strand of L-motif and the helix of S-motif together and is away from the putative CMP-Neu5Ac binding site (Figure 6C). Hence, mutation of either of these two residues is expected to destabilize the enzyme and consequently, lead to loss of activity. Structural/functional roles have also been deduced for other residues that are conserved in the SiaT superfamily based on the modeled 3-D structures; these deductions are in consonance with the results of experimental site-specific mutation studies (Table 2; Figure 6D).

### Relationship between family GT29 and family GT42 SiaTs

Eukaryotic [3-5] and prokaryotic [22-27] SiaTs have been classified into four families based on sequence similarity in the CAZy database [28]: (a) family GT29 contains viral and eukaryotic SiaTs; these enzymes have α2,3-, α2,6-, and α2,8-activities; (b) family GT38 contains bacterial polySiaTs mainly from *Escherichia coli *and *Neisseria meningitides*; (c) family GT42 contains SiaTs from *Campylobacter jejuni *and *Haemophilus influenzae *and (d) family GT52 contains α2,3-SiaT from *Neisseria gonorrhoeae, Neisseria meningitides *and few hypothetical SiaTs from *Haemophilus influenzae*. No sequence-based evolutionary relationship among these SiaT families has been established till date. Surprisingly, CstII was identified as the template for modeling the 3-D structures of human ST3Gals by fold-recognition servers; CstII belongs to family GT42 whereas human ST3Gals belong to family GT29. The modeled 3-D structures were found to be stereochemically acceptable and also were able to provide qualitative explanations for some of the site-specific mutagenesis data.

**Figure 7:**
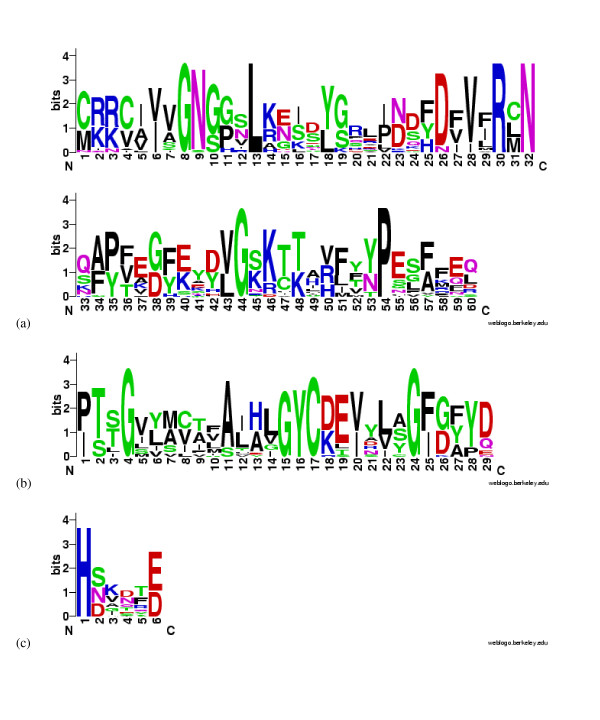
Sequence logos of (a) extended L-motif (b) S-motif (c) VS-motif. The conserved regions for generating the logos were extracted from the multiple sequence alignment of 23 experimentally characterized only α2,3-SiaTs from GT29 families and GT42 families (see Additional file 11). The multiple sequence alignment was generated using the FUGUE server. The colors used for the logos are as follows: blue, Lys, Arg and His; green, Cys, Ser, Gly, Thr and Tyr; red, Asp and Glu; pink, Asn and Gln; black, Ala, Val, Leu, Phe, Ile, Met, Pro and Trp.

The L-, S- and VS-motifs characteristic of mammalian SiaTs are thought to be absent in prokaryotic SiaTs [5]. The residues in CstII which correspond to these motif regions were identified by the structure-based sequence alignment generated by fold-recognition servers. A multiple sequence alignment of 14 experimentally characterized ST3Gal sequences (same as those in [11]) was submitted to the FUGUE server, which aligned these to CstII (Z score = 5.35). Using this alignment, multiple sequence alignments of experimentally characterized ST3Gals and family GT42 SiaTs were merged (see Additional file 11) and sequence logos were generated (Figure 7). Several residues in the L-, S- and VS-motif regions were found to be either strictly conserved or have conservative replacements in GT42 family SiaTs. This suggests that family GT42 SiaTs also have the L-, S- and VS-motifs (alignment positions 17–59, 165–189 and 225–230, respectively, in see Additional file 11). Conserved residues are found in other regions also (see Additional file 11). One such is the proline residue immediately after the L-motif (corresponding to position 54 in Figure 7); this residue is conserved in ST8 family also [11].

Family GT29 is actually a superfamily consisting of ST3Gal, ST6Gal, ST6GalNAc and ST8Sia families [5]. CstII was identified as the top hit by the fold-recognition server FFAS03 even for the human ST6Gal, ST6GalNAc and ST8Sia family members; the E-value in these cases is comparable to that obtained for ST3Gals. This suggests that other members of the GT29 family also share the CstII fold and thereby establish the structural similarities between GT29 and GT42 family members. On the contrary, CstII was not identified as a potential template when representative members of GT38 and GT52 families were submitted to FFAS03 server. This indicates the absence of any detectable structural similarities of GT38 and GT52 families with GT29 and GT42 family SiaTs.

## Conclusion

The knowledge of the 3-D structures of glycosyltransferases is important to better understand their biological function and to delineate structure-function relationships, as borne out, for example, in the case of galactosyltransferases [29-31]. This latter aspect is especially beneficial for the chemoenzymatic synthesis of carbohydrates and in turn, for glycomics (see, for example, [32]). SiaTs are another equally important class of glycosyltransferases but the 3-D structure for none of the human SiaTs is available till date. In light of these, the 3-D structure models of ST3Gals obtained in this study can be used to identify mutations that are likely to alter the donor and/or acceptor substrate specificities, thereby facilitating their use in the chemoenzymatic synthesis of complex carbohydrates and also to refine the predicted structures in the present study. This study has also provided another example of sequentially divergent proteins sharing a common fold to perform the same biochemical function.

## Methods

### Databases

The amino acid sequences of the experimentally characterized, human SiaTs belonging to the ST3Gal family (Table 1) were retrieved from the protein sequence database at NCBI . The 3-D structures of proteins were obtained from the protein data bank [33]. The fold classification of proteins is from the SCOP database [12,34].

### Servers

Protein sequence databases were searched using BLAST [35] or PSI-BLAST [36] servers at NCBI. FFAS03 [37], FUGUE [38], PHYRE (successor of 3D-PSSM, [39]), SAM-T02 [40] and GeneSilico Metaserver [41] were used for fold-recognition. Multiple sequence alignments were obtained using the TCoffee server [42,43]. Transmembrane helices were predicted using the TMHMM server v. 2.0 [44]. Secondary structures were predicted using the APSSP [45], JPRED [46], NNPREDICT [47], PROF [48], PSIPRED [49], SAM-T99 [50], SOPMA [51] and SSPRO [52] servers. Verify3D [53,54] and Colorado3D [55] were used to evaluate the models. DALI [56] and SSM [57] servers were used for 3-D structure comparisons. Sequence logos were created using *WebLogo *(version 2.8.1) [58]. All the servers were used with default values for the various parameters, except where mentioned otherwise.

### Software and hardware

BioEdit [59] was used for display and manipulation of sequences. SwissPDBviewer [60], Rasmol [61] and PyMol [62] were used to visualization and/or rendering. Modeller6v1, a homology modeling software, was used for modeling the 3-D structures [63,64]. The stereochemical quality of the generated model was assessed using PROCHECK [65,66]. All the software were run on an Intel Pentium IV desktop personal computer, except for modeller6v1, which was run on a SGI octane workstation. Default values were used for all the parameters, unless specified otherwise.

### Secondary structure prediction

The secondary structures of each of the six ST3Gals were predicted separately using eight prediction servers mentioned earlier. The secondary structures were predicted as three states, helix (H), strand (E) and coil (C). A consensus secondary structure was obtained by comparing the predictions of the eight servers. If different secondary structure states are predicted for a residue by the servers, the state that has been predicted by at least five of eight servers was taken as the consensus state; in other cases, it was marked as U (uncertain).

### Template-target sequence alignment

The ST3Gal sequences were submitted to fold-recognition servers separately. All the servers provide alignment of the submitted ST3Gal sequence (target) with the sequence of the potential hits (templates). Inspection of the template-target alignments generated by these fold-recognition servers revealed that certain regions of ST3Gals either did not have a template or the template-target secondary structures did not match. Such regions of ST3Gals were separately submitted to PSI-BLAST and fold-recognition servers. The best hits identified from these were then used as additional templates to model the target sequences.

### Validation of predicted 3-D structures

The stereochemical properties of predicted 3-D structures were assessed by PROCHECK and the residue environments by Verify3D and Colorado3D. Regions that are found by these servers as poorly modeled were improved by iterative manual adjustment of alignments and re-modeling. In the second stage of structure validation, the ability of the predicted structures to rationalize the results from the site-specific mutagenesis experiments reported in literature was investigated.

## Authors' contributions

MSS carried out the study and drafted the manuscript. PVB conceived the project, gave guidance and corrected the manuscript. Both MSS and PVB edited the final manuscript.

## Web links

**APSSP**: 

**BioEdit**: 

**BLAST server**: 

**Colorado3D**: 

**CAZy database**: 

**DALI**: 

**FFAS03**: 

**FUGUE**: 

**GeneSilico Metaserver**: 

**JPRED**: 

**MODELLER**: 

**NNPREDICT**: 

**PDB**: 

**PHYRE**: 

**PROCHECK**: 

**PROF**: 

**PSIPRED**: 

**PyMol: **

**RasMol**: 

**SAM-T02**: 

**SAM-T99**: 

**SCOP database**: 

**SOPMA**: 

**SSM**: 

**SSPRO**: 

**SwissPDBviewer**: 

**Tcoffee**: 

**TMHMM**: 

**Verify_3D**: 

**WebLogo**: 

## Supplementary Material

Additional File 1 Pairwise sequence similarity between sialyltransferases. These were calculated in BioEdit [59] using the pairwise global alignment option with the default BLOSUM62 matrix. The database accession numbers of the sequences are given in Table 1. The values above the shaded diagonal are for the complete sequences; the values below the diagonal are for the region from the L motif up to the C-terminus. Click here for file

Additional File 2 Consensus secondary structure derived from the predictions obtained from eight different servers for ST3Gal I (a), II (b), III (c), IV (d), V (e) and VI (f) sequences. Predictions from the eight servers agree with each other for 37–47% of residues in different SiaTs. At least five of the eight servers predict the same secondary structure for ~50% of the remaining residues and this was taken as the consensus secondary structure state. For the other 3–11% of residues, the secondary structure was noted as uncertain although some of these uncertainties can be resolved based on the secondary structure states of the flanking residues. Symbols H, E, C and U stand for helix, strand, coil and uncertain (See Methods) respectively. Click here for file

Additional File 3 Templates identified by fold-recognition servers for ST3Gals. The top hit alone is shown in each case. For each hit, the PDB code, subunit identifier, confidence score and the region of alignment (in the query sequence) are given. PDB id 1B37 is for polyamine oxidase, 1FC4 is for 2-amino-3-ketobutyrate CoA ligase, 1FIU is for restriction endonuclease NgoMIV from *Neisseria gonorrhoeae*, 1H7D is for aminolevulinic acid synthase 2, 1JF9 is for *Escherichia coli *selenocysteine lyase, 1K3R is for the hypothetical protein MT0001 from *Methanobacterium thermoautotrophicum*, 1KA1 is for PAPase Hal2p, 1R1G is for scorpion toxin BmBKTtx1 and 1RO7 is for sialyltransferase CstII from *Campylobacter jejuni*, 1W36 is for Recbcd DNA complex, 1W78 is for *Escherichia coli *FOLC. The interpretation of the confidence scores is as follows: **FUGUE **server: ZSCORE >= 6.0, certain (99% confidence); ZSCORE >= 4.0, likely (95% confidence); ZSCORE >= 3.5, marginal (90% confidence); ZSCORE >= 2.0, guess (50% confidence); ZSCORE < 2.0, uncertain. **FFAS03 **server: predictions with scores lower than -9.5 contain < 3% false positives. **SAM-T02 **server: E-value < ~1.0 × 10^-5 ^- very good hits; E-value > 0.1 - very speculative. **GeneSilico Metaserver**: pcons5 > 2.17 - reliable; pcons5 score > 1.03 but < 2.17 - unsure; pcons5 score < 1.03 - unreliable. ^¶^Templates were identified by submitting either the entire sequence or only the region from L motif up to the C-terminus. The L motif starts from residue 139 in ST3Gal I, 149 in ST3Gal II, 157 in ST3Gal III, 116 in ST3Gal IV, 136 in ST3Gal V and 115 in ST3Gal IV. Click here for file

Additional File 4 PDB IDs of templates that were used in addition to CstII for modeling ST3Gal structures. PDB Ids are from the protein databank [33]. Alignments are in Additional file 6. The proteins used are as follows: centromere Abp1 protein, IIUF; toxin Bmtx3, 1M2S; mechanosensitive channel protein Mscs, 1MXM; α-actinin 2, skeletal muscle isoform, 1H8B; cytochrome P450-terp, 1CPT; natural scorpion peptide P01, 1ACW; parathyroid hormone receptor, 1BL1; human S-adenosylmethionine decarboxylase, 1I7B; colicin D, 1V74; human defensin Hbd-2, 1E4Q; Fas death domain, 1DDF; topoisomerase I, 1YUA. Click here for file

Additional File 5 Schematic showing the domain architecture of ST3Gals and CstII. The transmembrane domain is at the N-terminus and the catalytic domain is at the C-terminus in ST3Gals. In contrast, the catalytic domain is at the N-terminus and the membrane-association region is at the C-terminus of CstII. Thus, the directions of the polypeptide chains in the region between the catalytic domain and transmembrane/membrane association regions in ST3Gal and CstII are opposite to each other. Click here for file

Additional File 6 Sequence alignments of ST3Gal I, II, III, IV, V, and VI (targets) with proteins of known 3-D structures (templates) used for modeling. The N-terminal region preceding the L-motif was modeled following the Cα-trace of the CstII C-terminus in reverse direction. The amino acid sequence of CstII in this region is italicized. 1RO7 was unanimously identified by FUGUE, FFAS03 and SAM-T02 as the template for all the ST3Gals. The names of other templates are given in the footnote to the Table in Additional file 4. The numbers at the top correspond to the sequence number of the appropriate ST3Gal. The characters above the ST3Gal sequences indicate helices and strands (nomenclature as in Figure 1); the letters H (for helix) and E (for strand) at the bottom of each block of alignment indicate the secondary structure in the template. Click here for file

Additional File 7 Stereo chemical qualities of the generated models. The values are for the top three models of ST3Gals except ST3Gal VI, for which the values are reported for the top four models. The average score per residue with different window sizes were calculated using the Colorado3D server. The range of scores obtained for the 25 models obtained using Modeller, Procheck and Verify3D are reported for CstII. The modeling was done by aligning the CstII sequence with its own structure (PDB id 1RO7). Click here for file

Additional File 8 3-D structures of CstII (template) and modeled ST3Gals with residues color-coded based on ProsaII scores and rendered using SwissPDBViewer. The ProsaII scores were obtained using the Colorado3D server. Blue regions indicate good scores and red indicate bad scores. The rendering for CstII (*top row*) shows the superposition of all the 25 models generated. A representative structure from among the top three/four models is shown for ST3Gal I, II, and III (*middle row, from left to right*) and ST3Gal IV, V and VI (*bottom row, from left to right*). The average scores per residue obtained using window size 5 are as follows: -1.34, CstII; -0.07, ST3Gal I; -0.19, ST3Gal II; 0.2, ST3Gal III; -0.25, ST3Gal IV; 0.03, ST3Gal V; -0.02, ST3Gal IV. Click here for file

Additional File 9 3-D structures of CstII (template) and modeled ST3Gals with residues color-coded based on Verify3D scores and rendered using SwissPDBViewer. The Verify3D scores were obtained using the Colorado3D server. Blue regions indicate good scores and red indicate bad scores. The rendering for CstII (*top row*) shows the superposition of all the 25 models generated. A representative structure from among the top three/four models is shown for ST3Gal I, II, and III (*middle row, from left to right*) and ST3Gal IV, V and VI (*bottom row, from left to right*). The average scores per residue obtained using window size 5 are as follows: 0.46, CstII; 0.27, ST3Gal I; 0.26, ST3Gal II; 0.22, ST3Gal III; 0.27, ST3Gal IV; 0.26, ST3Gal V; 0.22, ST3Gal VI. Click here for file

Additional File 10 Structural comparisons between the modeled ST3Gals and CstII. The SSM [57] and DALI [56] servers were used for structure comparison. Both the servers identify CstII as the top hit. *RMSD *represents root mean square deviation calculated between Cα-atoms of matched residues at best 3D superposition of the query and target structures. *Nalign *represents the number of matched residues between the query and target. *Qscore *is a quality function of Cα-alignment. It's a combined parameter for *Nalign *and *RMSD*. The identical structures have a *Qscore *of 1. *Zscore *is a statistical significance score for best domain-domain alignment. Click here for file

Additional File 11 Multiple sequence alignment of experimentally characterized mammalian α2,3-SiaTs and SiaTs from GT42 family. Only the experimentally characterized SiaT sequences have been taken from both the families. The sequence alignment was generated by first aligning mammalian SiaTs with CstII using FUGUE; this was used as guide to merge the multiple sequence alignments of mammalian ST3Gals and GT42 family members. The L-, S- and VS-motif regions have been marked in the alignment. The regions of protein marked in red were used to generate the sequence logos (Figure 7). The sequences used are: bifunctional α2,3/8-sialyltransferase, CstII (1RO7); α2,3-sialyltransferase, CstI (AAF13495); α2,3-sialyltransferase, CstII (AAF34137); bifunctional α2,3/8-sialyltransferase, CstII (AAL06004); α2,3-sialyltransferase, CstI (AAF13495); α2,3-sialyltransferase, CstII (AAF34137); bifunctional α2,3/8-sialyltransferase, CstII (AAL06004), α2,3-sialyltransferase, CstIII (AAK73183); α2,3/8-sialyltransferase, CstII (AAF31771); all these proteins are from *Campylobacter jejuni*. The experimentally characterized mammalian α2,3 sialyltransferases are taken from [11]. ST3Gal I (Q11201, human; P54751, mouse; Q11200, chick; Q02745, pig), ST3Gal II (Q16842, human; NP_835149, mouse), ST3Gal III (Q11203, human; P97325, mouse and Q02734, rat), ST3Gal IV (Q11206, human; NP_033204, mouse), ST3Gal V (Q9UNP4, human; O88829, mouse) and ST3Gal VI (Q9Y274, human). The H. influenzae sequences in family GT42 have not been used in the multiple sequence alignment, and hence to generate sequence logos, because all these SiaTs are computationally annotated sequences. Click here for file

## Figures and Tables

**Table 1:** Accession numbers and modelled regions of human SiaTs

**Name**	**NCBI accession number**	**Length (number of residues)**	**Transmembrane domain**^¶^	**Modeled region**^§^	**Reference**
ST3Gal I	AAA36612	340	12–35	66–332	[63]
ST3Gal II	NP_008858	350	7–29	76–347	[64]
ST3Gal III	AAA35778	375	4–26	92–369	[65]
ST3Gal IV	AAA16460	332	5–20	51–329	[66]
ST3Gal V	Q9UNP4	362	15–32	70–354	[67]
ST3Gal VI	Q9Y274	331	10–32	51–323	[19]

^¶^Predicted using the TMHMM server [42].

^§^All the residues of the protein except those in the N-terminal cytoplasmic, transmembrane domains and last few residues of the catalytic region were considered for 3-D structure modeling. However, part of the neck/stem region could not be modeled for reasons detailed in the text.

**Table 2:** Roles deduced for some of the residues which are conserved in the eukaryotic SiaT superfamily and whose mutations have been experimentally characterized^#^

**Mutant**	**Equivalent residue in ST3Gal I**	**K**_m_**(μM) Donor/Acceptor**	**Activity**	Role deduced for the mutated residue from the modeled 3-D structures^‡^
Mutations in L motif^¶^				

Wild type		50/331	100%	
C181A	C142	-/-	< 5%	Structural role: involved in disulphide bridge
V184A	V145	300/372	~45%	Structural role: part of hydrophobic core^†^
L190A	L151	597/188	~28%	Structural role: part of hydrophobic core^†^
R207A	R168	-/-	< 5%	Structural role: is buried and hydrogen bonds with side chains of N147 and E178 (ST3Gal I numbering). N147 is replaced by Ser in ST3Gal V, but Ser does not form hydrogen bond with Arg
V220A	V181	343/260	30%	Structural role: part of the hydrophobic core^†^
S222A	T183	-/-	< 5%	Structural role: These are at beginning of strand β4 and are solvent exposed
K223A	K184	330/400	32%	
T225A	T186	160/200	27%	
Mutations in S motif^§^				
Wild type		50/330	100	
P318A	P267	-/-	very low	Structural role: helix J nucleator
S319A	S268	-/-	< 5%	Functional role: close to ribose
C332A	C281	-/-	< 5%	Structural role: forms disulphide bridge
V335L	V284	80/322	79%	Structural role: part of the hydrophobic core
V335A	V284	89/947	83%	
Mutations in motif-III*				
H299A		-/-	loss of activity	Functional role: close to phosphate
Y300A		-/-		

^#^Residues that are either strictly conserved or have conservative replacements in the eukaryotic SiaT superfamily are from [11].

^¶^Data corresponds to mutants of ST6Gal I [8]. Hyphen (" - ") indicates values could not be determined.

^§^Data corresponds to mutants of ST6Gal I [9]. Hyphen (" - ") indicates values could not be determined.

*Data corresponds to mutants of ST3Gal I [7]. Hyphen (" - ") indicates values could not be determined.

^‡^The mutation is considered structural if it destabilizes the structure and is far from functional site and it is termed functional if it is part of the substrate binding site.

^†^Are part of the same hydrophobic core.
