# Supplementary material for: Fold-recognition and comparative modeling of human α2,3-sialyltransferases reveal their sequence and structural similarities to CstII from Campylobacter jejuni
Source: BMC Struct Biol. 2006 Apr 19;6:9. doi: 10.1186/1472-6807-6-9 (PMC1508147; doi:10.1186/1472-6807-6-9)
Supplement: Additional File 1 — Pairwise sequence similarity between sialyltransferases. These were calculated in BioEdit [59] using the pairwise global alignment option with the default BLOSUM62 matrix. The database accession numbers of the sequences are given in Table 1. The values above the shaded diagonal are for the complete sequences; the values below the diagonal are for the region from the L motif up to the C-terminus. [file 1472-6807-6-9-S1.DOC]

|  | ST3Gal I | ST3Gal II | ST3Gal III | ST3Gal IV | ST3Gal V | ST3Gal VI |
| --- | --- | --- | --- | --- | --- | --- |
| ST3Gal I |  | 66 | 45 | 45 | 42 | 42 |
| ST3Gal II | 80 |  | 43 | 44 | 41 | 42 |
| ST3Gal III | 50 | 49 |  | 51 | 54 | 51 |
| ST3Gal IV | 51 | 50 | 61 |  | 51 | 54 |
| ST3Gal V | 45 | 46 | 65 | 58 |  | 49 |
| ST3Gal VI | 46 | 48 | 63 | 64 | 57 |  |
