# Supplementary material for: Fold-recognition and comparative modeling of human α2,3-sialyltransferases reveal their sequence and structural similarities to CstII from Campylobacter jejuni
Source: BMC Struct Biol. 2006 Apr 19;6:9. doi: 10.1186/1472-6807-6-9 (PMC1508147; doi:10.1186/1472-6807-6-9)
Supplement: Additional File 2 — Consensus secondary structure derived from the predictions obtained from eight different servers for ST3Gal I (a), II (b), III (c), IV (d), V (e) and VI (f) sequences. Predictions from the eight servers agree with each other for 37–47% of residues in different SiaTs. At least five of the eight servers predict the same secondary structure for ~50% of the remaining residues and this was taken as the consensus secondary structure state. For the other 3–11% of residues, the secondary structure was noted as uncertain although some of these uncertainties can be resolved based on the secondary structure states of the flanking residues. Symbols H, E, C and U stand for helix, strand, coil and uncertain (See Methods) respectively. [file 1472-6807-6-9-S2.doc]

(a) Consensus secondary structure of ST3Gal I

10 20 30 40 50 60 70

....|....| ....|....| ....|....| ....|....| ....|....| ....|....| ....|....|

MVTLRKRTLK VVTFLVLFIF LTSFFLNYSH TMVATTWFPK QMVLELSENL KRLIKHRPCT CTHCIGQRKL

CCUUUHHHHH HHHHHHHHHH HHHHHUHCCC CCCCCCCCCU UUHUHUHHHH HHHHCCCCCC CCCCHUUHCH

80 90 100 110 120 130 140

....|....| ....|....| ....|....| ....|....| ....|....| ....|....| ....|....|

SAWFDERFNQ TMQPLLTAQN ALLEDDTYRW WLRLQREKKP NNLNDTIKEL FRVVPGNVDP MLEKRSVGCR

HHHHHHHCCC CCCCUUCCCC CCCCCCCUHH UUUUCCCCCC CCCUUHHHHH HHHCCCCCCC CCCCCCCCCC

150 160 170 180 190 200 210

....|....| ....|....| ....|....| ....|....| ....|....| ....|....| ....|....|

RCAVVGNSGN LRESSYGPEI DSHDFVLRMN KAPTAGFEAD VGTKTTHHLV YPESFRELGD NVSMILVPFK

EEEEEECCCC CCCCCCCCCC CCCCEEEEEC CCCCCCUUUC CCCCCEEEEE CCCHHHHCCC CCEEEEECCC

220 230 240 250 260 270 280

....|....| ....|....| ....|....| ....|....| ....|....| ....|....| ....|....|

TIDLEWVVSA ITTGTISHTY IPVPAKIRVK QDKILIYHPA FIKYVFDNWL QGHGRYPSTG ILSVIFSMHV

CCCHHHUHHH HUCCCCCCCC CCCCCCCCCC CCCEEEECUH HHHHHHHHHH HCCCCCCCCH HHHHHHHHHH

290 300 310 320 330 340

....|....| ....|....| ....|....| ....|....| ....|....| ....|....|

CDEVDLYGFG ADSKGNWHHY WENNPSAGAF RKTGVHDADF ESNVTATLAS INKIRIFKGR

CCCEEEEEEC CCCCCCCEEE ECCCCCCCUU CCCCCCCCHH HHHHHHHHHH UCCEEEECCC

(b) Consensus secondary structure of ST3Gal II

10 20 30 40 50 60 70

....|....| ....|....| ....|....| ....|....| ....|....| ....|....| ....|....|

MKCSLRVWFL SVAFLLVFIM SLLFTYSHHS MATLPYLDSG ALDGTHRVKL VPGYAGLQRL SKERLSGKSC

CCCUUHHHHH HHHHHHHHHH HHHHUHHHCC CCCCCCCCCC CCCCCCUUUU CCCCCUHHHH CCCCCCCCCC

80 90 100 110 120 130 140

....|....| ....|....| ....|....| ....|....| ....|....| ....|....| ....|....|

ACRRCMGDAG ASDWFDSHFD GNISPVWTRE NMDLPPDVQR WWMMLQPQFK SHNTNEVLEK LFQIVPGENP

CCCCUHUUUC CCHHHHHUCC CCCCUUUCCC CCCCCCCUHH HHHHUCCCCC CCCUHHHHHH HHHHCCCCCC

150 160 170 180 190 200 210

....|....| ....|....| ....|....| ....|....| ....|....| ....|....| ....|....|

YRFRDPHQCR RCAVVGNSGN LRGSGYGQDV DGHNFIMRMN QAPTVGFEQD VGSRTTHHFM YPESAKNLPA

CCCCCCCCCC EEEEEECCCC CCCCCCCCCC CCCCEEEEEC CCCCCCCUUC CCCCCEEEEE CCCUHUUCCC

220 230 240 250 260 270 280

....|....| ....|....| ....|....| ....|....| ....|....| ....|....| ....|....|

NVSFVLVPFK VLDLLWIASA LSTGQIRFTY APVKSFLRVD KEKVQIYNPA FFKYIHDRWT EHHGRYPSTG

CCEEEEECCC CCUHHHHHHH HHCCCCCEEU CCCCCUUCCC CCCEEEUCUH HHHHHHHHHH UCCCCCCCUH

290 300 310 320 330 340 350

....|....| ....|....| ....|....| ....|....| ....|....| ....|....| ....|....|

MLVLFFALHV CDEVNVYGFG ADSRGNWHHY WENNRYAGEF RKTGVHDADF EAHIIDMLAK ASKIEVYRGN

HHHHHHHHHH CCCEEEEEEC CCCCCCCUEE ECCCCCCCUU CCCCCCCCHH HHHHHHHHHH CCCEEEECCC

(c) Consensus secondary structure of ST3Gal III

10 20 30 40 50 60 70

....|....| ....|....| ....|....| ....|....| ....|....| ....|....| ....|....|

MGLLVFVRNL LLALCLFLVL GFLYYSAWKL HLLQWEEDSN SVVLSFDSAG QTLGSEYDRL GFLLNLDSKL

CCUHHHHHHH HHHHHHHHHH HHHHHHHHHH HHUUCCCCCC CUUUUUCCCC CCCCCHHHHH HHHUCCCCCC

80 90 100 110 120 130 140

....|....| ....|....| ....|....| ....|....| ....|....| ....|....| ....|....|

PAELATKYAN FSEGACKPGY ASALMTAIFP RFSKPAPMFL DDSFRKWARI REFVPPFGIK GQDNLIKAIL

CHHHHHHHHU CCCCCCCCCH HHHHHHHHCC CCCCCCCCCC CCCHHHHHHH UCCCCCCCCC CUHHHHHHHH

150 160 170 180 190 200 210

....|....| ....|....| ....|....| ....|....| ....|....| ....|....| ....|....|

SVTKEYRLTP ALDSLRCRRC IIVGNGGVLA NKSLGSRIDD YDIVVRLNSA PVKGFEKDVG SKTTLRITYP

HHCCCCCCCC CCCCCCCCEE EEECCCCUUC CCCCCCCCCC CEEEEEECCC CCCCCUUCCC CCCEEEEECC

220 230 240 250 260 270 280

....|....| ....|....| ....|....| ....|....| ....|....| ....|....| ....|....|

EGAMQRPEQY ERDSLFVLAG FKWQDFKWLK YIVYKERVSA SDGFWKSVAT RVPKEPPEIR ILNPYFIQEA

CCCCCCCCCC CCCCEEEEEC CCCCCHHHHH HHHHCCCCCC CCCCCCCCCC CCCCCCCCEE ECCCHHHHHH

290 300 310 320 330 340 350

....|....| ....|....| ....|....| ....|....| ....|....| ....|....| ....|....|

AFTLIGLPFN NGLMGRGNIP TLGSVAVTMA LHGCDEVAVA GFGYDMSTPN APLHYYETVR MAAIKESWTH

HHHHHUCCCC CCCCCCCCCC CCHHHHHHHH HHHCCUEEEE ECCCCCCCCC CCUEECCCCU HHHHHCCCCC

360 370

....|....| ....|....| ....|

NIQREKEFLR KLVKARVITD LSSGI

CCHHHHHHHH HHHHCCCUEU CCCCC

(d) Consensus secondary structure of ST3Gal IV

10 20 30 40 50 60 70

....|....| ....|....| ....|....| ....|....| ....|....| ....|....| ....|....|

MCPAGWKLLA MLALVLVVMV WYSISREDRY IELFYFPIPE KKEPCLQGEA ESKASKLFGN YSRDQPIFLR

CCCCUHHHHH HHHHHHHHHH HHUUCCCCCU UUUUCCCCCC CCCCCCCUHH HHHHHHHHUU CCCCCCUUUC

80 90 100 110 120 130 140

....|....| ....|....| ....|....| ....|....| ....|....| ....|....| ....|....|

LEDYFWVKTP SAYELPYGTK GSEDLLLRVL AITSSSIPKN IQSLRCRRCV VVGNGHRLRN SSLGDAINKY

CCCHUUUHUU CCCCCCCCCC CCHHHHHHHH UCCCCCCCCC CCCCCCCEEE EEECCCCUCC CCCCCCCCCC

150 160 170 180 190 200 210

....|....| ....|....| ....|....| ....|....| ....|....| ....|....| ....|....|

DVVIRLNNAP VAGYEGDVGS KTTMRLFYPE SAHFDPKVEN NPDTLLVLVA FKAMDFHWIE TILSDKKRVR

UEEEEECCCC CCCCUUCCCC CEEEEEECCC CCCCCCCCCC CCCCEEEEEU CCCCCHHHHH HHHHCCCCCC

220 230 240 250 260 270 280

....|....| ....|....| ....|....| ....|....| ....|....| ....|....| ....|....|

KGFWKQPPLI WDVNPKQIRI LNPFFMEIAA DKLLSLPMQQ PRKIKQKPTT GLLAITLALH LCDLVHIAGF

CCCCCCCCCC UCCCCCUEEE CCHHHHHHHH HHHHUCCCCC CCCCCCCCCH HHHHHHHHHH HCCUEEEEEU

290 300 310 320 330

....|....| ....|....| ....|....| ....|....| ....|....| ..

GYPDAYNKKQ TIHYYEQITL KSMAGSGHNV SQEALAIKRM LEMGAIKNLT SF

CCCCCCCCCC CEEEUCCCCH HHHUCCCCCU HHHHHHHHHH HHHCCCUUCC CC

(e) Consensus secondary structure of ST3Gal V

10 20 30 40 50 60 70

....|....| ....|....| ....|....| ....|....| ....|....| ....|....| ....|....|

MRRPSLLLKD ILKCTLLVFG VWILYILKLN YTTEECDMKK MHYVDPDRVK RAQKYAQQVL QKECRPKFAK

CCCCUHHHHH HHHHHHHHHH HHHHHUUUCC CCCCCUUUUU CCCCCCCCUU HHHHUUCCCC CCCCCCHHHH

80 90 100 110 120 130 140

....|....| ....|....| ....|....| ....|....| ....|....| ....|....| ....|....|

TSMALLFEHR YSVDLLPFVQ KAPKDSEAES KYDPPFGFRK FSSKVQTLLE LLPEHDLPEH LKAKTCRRCV

HHHHHHHHCC CCCCCCUHHC CCCCCCHHHU CCCCCCCCCC HHHHHHHHHH HCCCCCCCCC CCCCCCCEEE

150 160 170 180 190 200 210

....|....| ....|....| ....|....| ....|....| ....|....| ....|....| ....|....|

VIGSGGILHG LELGHTLNQF DVVIRLNSAP VEGYSEHVGN KTTIRMTYPE GAPLSDLEYY SNDLFVAVLF

EEECCCCUCC CCCCCCCCCC UEEEEECCCC CCCCUUCCCC CEEEEEECCC CCCCCCUUCC CCCEEEEEEC

220 230 240 250 260 270 280

....|....| ....|....| ....|....| ....|....| ....|....| ....|....| ....|....|

KSVDFNWLQA MVKKETLPFW VRLFFWKQVA EKIPLQPKHF RILNPVIIKE TAFDILQYSE PQSRFWGRDK

CCCCHHHHHH HHHCCCCCUC CCCCCCCCCC CCCCCCCCCE EEUCCHHHHH HHHHHHHCCC CCCCCCCCCC

290 300 310 320 330 340 350

....|....| ....|....| ....|....| ....|....| ....|....| ....|....| ....|....|

NVPTIGVIAV VLATHLCDEV SLAGFGYDLN QPRTPLHYFD SQCMAAMNFQ TMHNVTTETK FLLKLVKEGV

CCCCUHHHHH HHHHHHCCUE EEEEUCCCCC CCCCCUEECC CCCUHHHHHC CCCCUHHHHH HHHHHHHCCC

360

....|....| ..

VKDLSGGIDR EF

UUUUCCCCCC CC

(f) Consensus secondary structure of ST3Gal VI

10 20 30 40 50 60 70

....|....| ....|....| ....|....| ....|....| ....|....| ....|....| ....|....|

MRGYLVAIFL SAVFLYYVLH CILWGTNVYW VAPVEMKRRN KIQPCLSKPA FASLLRFHQF HPFLCAADFR

CCCUUHHHHH HHHHHHHHHH HHUCCCCCEE ECCUUUCCCC CCCCCCCCCC HHHHHHCCCC CCCUCCCCCC

80 90 100 110 120 130 140

....|....| ....|....| ....|....| ....|....| ....|....| ....|....| ....|....|

KIASLYGSDK FDLPYGMRTS AEYFRLALSK LQSCDLFDEF DNIPCKKCVV VGNGGVLKNK TLGEKIDSYD

UHHHHCCCCC CCCCCCCCCH HHHHHHHHHU CCCCCCCCCC CCCCCCEEEE EECCCCUCCC CCCCCCCCCU

150 160 170 180 190 200 210

....|....| ....|....| ....|....| ....|....| ....|....| ....|....| ....|....|

VIIRMNNGPV LGHEEEVGRR TTFRLFYPES VFSDPIHNDP NTTVILTAFK PHDLRWLLEL LMGDKINTNG

EEEEECCCCC CCCUUCCCCC UEEEEECCCC CCCCCCCCCC CCEEEEEECC CCCHHHHHHH HUCCCCCCCC

220 230 240 250 260 270 280

....|....| ....|....| ....|....| ....|....| ....|....| ....|....| ....|....|

FWKKPALNLI YKPYQIRILD PFIIRTAAYE LLHFPKVFPK NQKPKHPTTG IIAITLAFYI CHEVHLAGFK

CCCCCCCCCC CCCCCEEECC UHHHHHHHHH HHUCCCCCCC CCCCCCCCCH HHHHHHHHHH CCCEEEEEUC

290 300 310 320 330

....|....| ....|....| ....|....| ....|....| ....|....| .

YNFSDLKSPL HYYGNATMSL MNKNAYHNVT AEQLFLKDII EKNLVINLTQ D

CCCCCCCCCC EEUCCCCCHH HCCCCCCCHH HHHHHHHHHH HHCCUUUCCC C
