# Supplementary material for: Fold-recognition and comparative modeling of human α2,3-sialyltransferases reveal their sequence and structural similarities to CstII from Campylobacter jejuni
Source: BMC Struct Biol. 2006 Apr 19;6:9. doi: 10.1186/1472-6807-6-9 (PMC1508147; doi:10.1186/1472-6807-6-9)
Supplement: Additional File 3 — Templates identified by fold-recognition servers for ST3Gals. The top hit alone is shown in each case. For each hit, the PDB code, subunit identifier, confidence score and the region of alignment (in the query sequence) are given. PDB id 1B37 is for polyamine oxidase, 1FC4 is for 2-amino-3-ketobutyrate CoA ligase, 1FIU is for restriction endonuclease NgoMIV from Neisseria gonorrhoeae, 1H7D is for aminolevulinic acid synthase 2, 1JF9 is for Escherichia coli selenocysteine lyase, 1K3R is for the hypothetical protein MT0001 from Methanobacterium thermoautotrophicum, 1KA1 is for PAPase Hal2p, 1R1G is for scorpion toxin BmBKTtx1 and 1RO7 is for sialyltransferase CstII from Campylobacter jejuni, 1W36 is for Recbcd DNA complex, 1W78 is for Escherichia coli FOLC. The interpretation of the confidence scores is as follows: FUGUE server: ZSCORE >= 6.0, certain (99% confidence); ZSCORE >= 4.0, likely (95% confidence); ZSCORE >= 3.5, marginal (90% confidence); ZSCORE >= 2.0, guess (50% confidence); ZSCORE < 2.0, uncertain. FFAS03 server: predictions with scores lower than -9.5 contain < 3% false positives. SAM-T02 server: E-value < ~1.0 × 10^-5^ - very good hits; E-value > 0.1 - very speculative. GeneSilico Metaserver: pcons5 > 2.17 - reliable; pcons5 score > 1.03 but < 2.17 - unsure; pcons5 score < 1.03 - unreliable. ¶Templates were identified by submitting either the entire sequence or only the region from L motif up to the C-terminus. The L motif starts from residue 139 in ST3Gal I, 149 in ST3Gal II, 157 in ST3Gal III, 116 in ST3Gal IV, 136 in ST3Gal V and 115 in ST3Gal IV. [file 1472-6807-6-9-S3.doc]

|  | **Complete ST3Gal Sequence^¶^** | | | | **ST3Gal Sequence from L motif^¶^** | | | |
| --- | --- | --- | --- | --- | --- | --- | --- | --- |
|  | **Meta** | **FUGUE** | **FFAS03** | **SAM-T02** | **Meta** | **FUGUE** | **FFAS03** | **SAM-T02** |
| ST3Gal I | 1B37 A,  0.27,  1-326 | 1H7D A,  3.1,  132-182 | 1RO7 A,  –11.4,  139-337 | 1RO7 A,  0.62,  140-337 | 1RO7 A,  0.54,  139-337 | 1RO7 A,  8.31,  140-340 | 1RO7 A,  –12.5,  139-337 | 1RO7 A,  0.019,  141 - 338 |
| ST3Gal II | 1B37 A,  0.47,  1-336 | 1K3R A  3.25,  9-346 | 1RO7 A,  -12.4,  149-347 | 1RO7 A,  2.8,  150-347 | 1RO7 A,  0.53,  149-347 | 1RO7 A,  6.6,  150-350 | 1RO7 A,  –13.1,  149-347 | 1RO7 A,  0.03,  151 - 348 |
| ST3Gal III | 1KA1 A,  0.19,  1-375 | 1FIU A,  2.13,  20-375 | 1RO7 A,  –11.1,  158-372 | 1RO7 A,  13.0,  159-371 | 1RO7 A,  0.43,  158-372 | 1RO7 A,  5.23,  158-375 | 1RO7 A,  –12.7,  158-372 | 1RO7 A,  0.22,  159 - 358 |
| ST3Gal IV | 1W78 A,  0.30,  1-325 | 1H7D A,  3.89,  109-160 | 1RO7 A,  –10.4,  116-329 | 1RO7 A,  1.5,  117-329 | 1RO7 A,  0.47,  116-329 | 1RO7 A,  5.48,  117-332 | 1RO7 A,  –11.1,  116-329 | 1RO7 A,  0.12,  118 - 328 |
| ST3Gal V | 1FC4 A,  0.22,  1-352 | 1R1G A,  2.77,  130-154 | 1RO7 A,  –11.8,  136-354 | 1JF9 A  27.0,  274-295 | 1RO7 A,  0.52,  136-360 | 1RO7 A,  5.83,  137-362 | 1RO7 A,  –11.7,  136-354 | 1RO7 A,  0.26,  138 - 355 |
| ST3Gal VI | 1W36 D,  0.21,  1-332 | 1H7D A,  3.61,  110-159 | 1RO7 A,  –10.8,  115-326 | 1RO7 A,  3.0,  116-328 | 1RO7 A,  0.35,  115-326 | 1RO7 A,  3.74,  116-331 | 1RO7 A,  –10.8,  115-326 | 1RO7 A,  0.069,  117 - 329 |
