# Supplementary material for: Fold-recognition and comparative modeling of human α2,3-sialyltransferases reveal their sequence and structural similarities to CstII from Campylobacter jejuni
Source: BMC Struct Biol. 2006 Apr 19;6:9. doi: 10.1186/1472-6807-6-9 (PMC1508147; doi:10.1186/1472-6807-6-9)
Supplement: Additional File 4 — PDB IDs of templates that were used in addition to CstII for modeling ST3Gal structures. PDB Ids are from the protein databank [33]. Alignments are in Additional file 6. The proteins used are as follows: centromere Abp1 protein, IIUF; toxin Bmtx3, 1M2S; mechanosensitive channel protein Mscs, 1MXM; α-actinin 2, skeletal muscle isoform, 1H8B; cytochrome P450-terp, 1CPT; natural scorpion peptide P01, 1ACW; parathyroid hormone receptor, 1BL1; human S-adenosylmethionine decarboxylase, 1I7B; colicin D, 1V74; human defensin Hbd-2, 1E4Q; Fas death domain, 1DDF; topoisomerase I, 1YUA. [file 1472-6807-6-9-S4.doc]

| Target Protein | Region of Target Protein^§^ | | |
| --- | --- | --- | --- |
|  | Region preceding L-motif | Helix C to β6 | Helix E to F |
| ST3Gal I | 1IUF | 1M2S and 1MXM | - |
| ST3Gal II | 1IUF and 1H8B | 1M2S, 1ACW and 1CPT | - |
| ST3Gal III | 1BL1 and 1I7B | 1V74 | 1E4Q |
| ST3Gal IV | 1BL1 and 1I7B | 1V74 | 1E4Q |
| ST3Gal V | 1BL1 and 1I7B | 1V74 | 1E4Q |
| ST3Gal VI | 1DDF | 1V74 | 1YUA |
