# Supplementary figures and images for: Fold-recognition and comparative modeling of human α2,3-sialyltransferases reveal their sequence and structural similarities to CstII from Campylobacter jejuni
Source: BMC Struct Biol. 2006 Apr 19;6:9. doi: 10.1186/1472-6807-6-9 (PMC1508147; doi:10.1186/1472-6807-6-9)

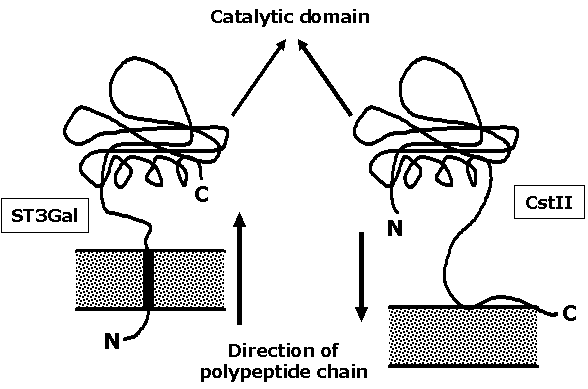

Supplement: Additional File 5 — Schematic showing the domain architecture of ST3Gals and CstII. The transmembrane domain is at the N-terminus and the catalytic domain is at the C-terminus in ST3Gals. In contrast, the catalytic domain is at the N-terminus and the membrane-association region is at the C-terminus of CstII. Thus, the directions of the polypeptide chains in the region between the catalytic domain and transmembrane/membrane association regions in ST3Gal and CstII are opposite to each other. [file 1472-6807-6-9-S5.doc]

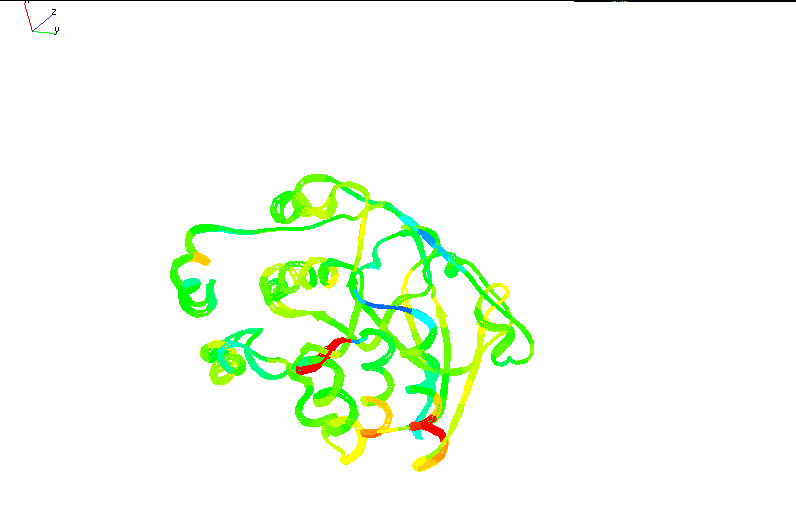


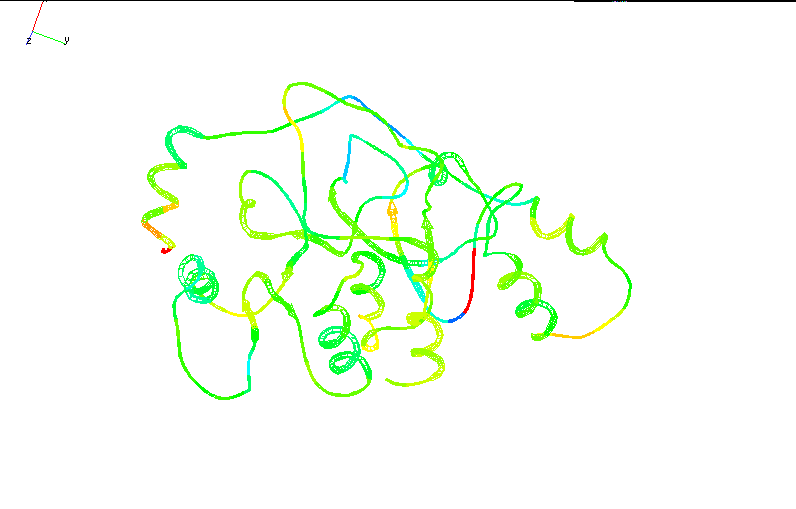

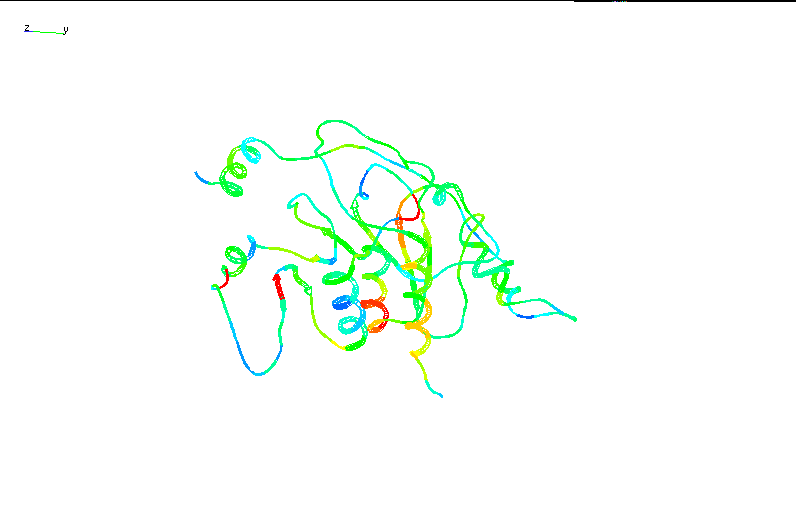

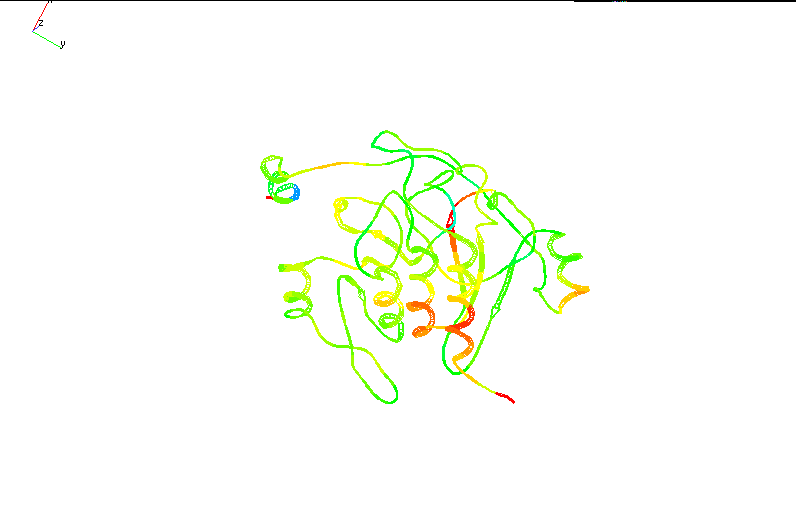


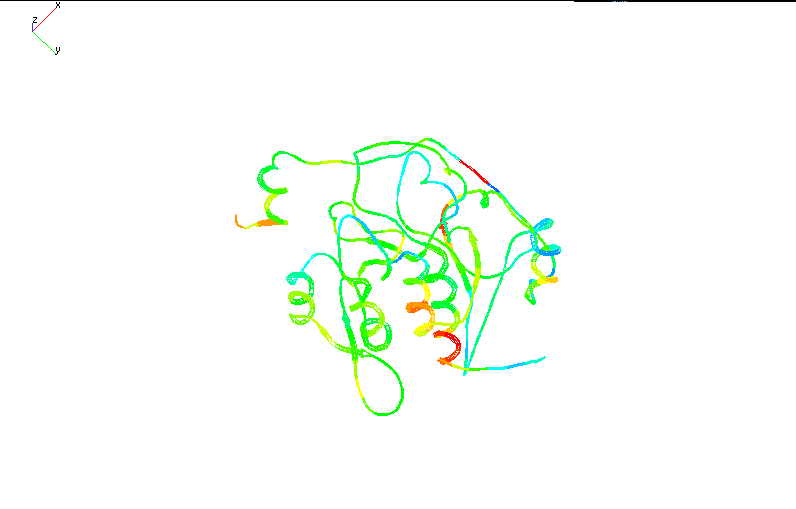

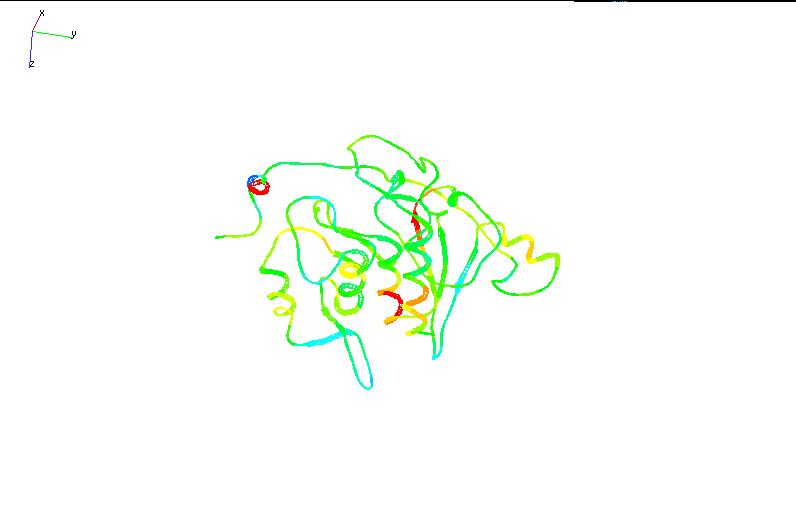

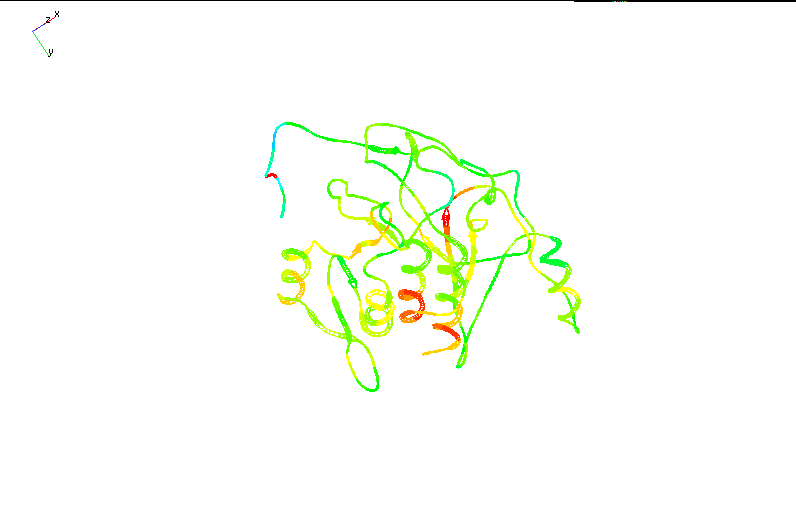

Supplement: Additional File 8 — 3-D structures of CstII (template) and modeled ST3Gals with residues color-coded based on ProsaII scores and rendered using SwissPDBViewer. The ProsaII scores were obtained using the Colorado3D server. Blue regions indicate good scores and red indicate bad scores. The rendering for CstII (top row) shows the superposition of all the 25 models generated. A representative structure from among the top three/four models is shown for ST3Gal I, II, and III (middle row, from left to right) and ST3Gal IV, V and VI (bottom row, from left to right). The average scores per residue obtained using window size 5 are as follows: -1.34, CstII; -0.07, ST3Gal I; -0.19, ST3Gal II; 0.2, ST3Gal III; -0.25, ST3Gal IV; 0.03, ST3Gal V; -0.02, ST3Gal IV. [file 1472-6807-6-9-S8.doc]

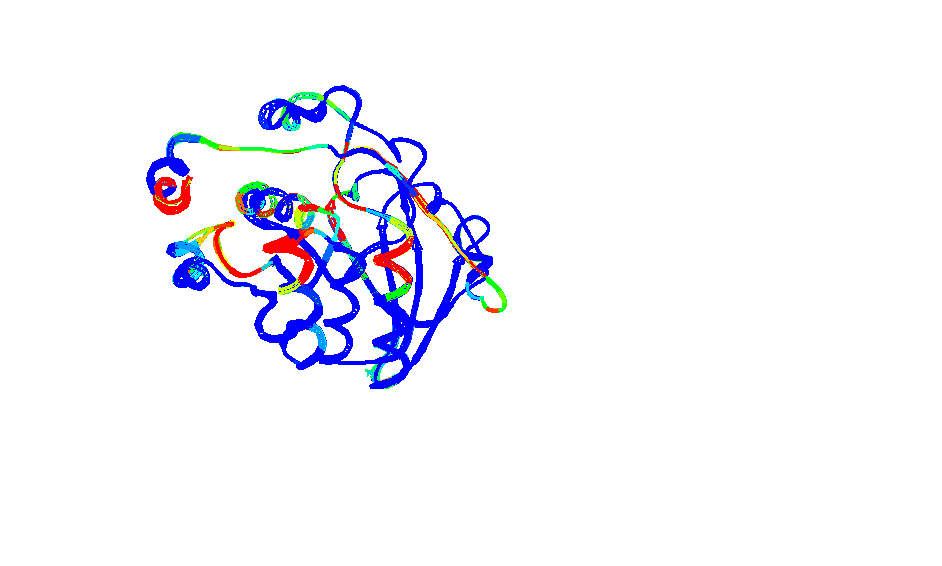


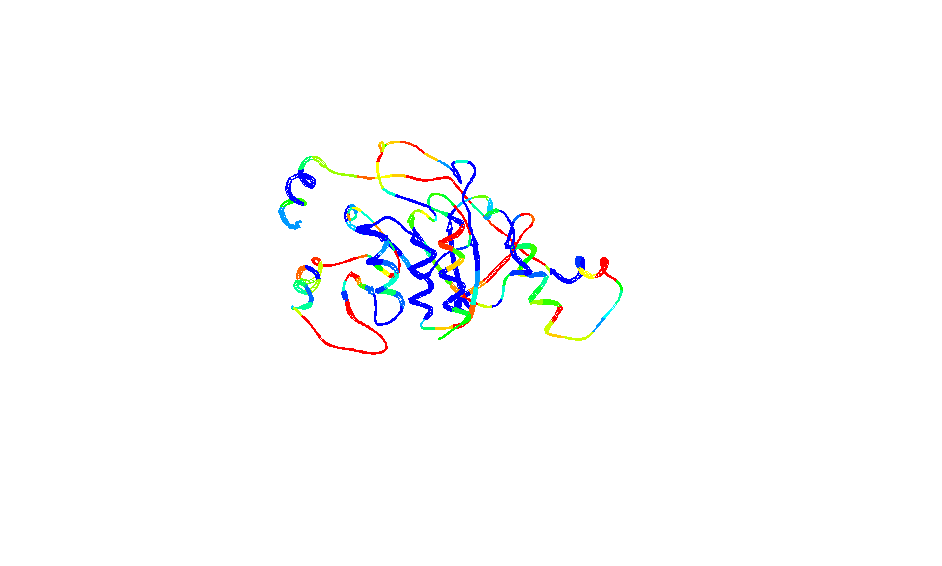

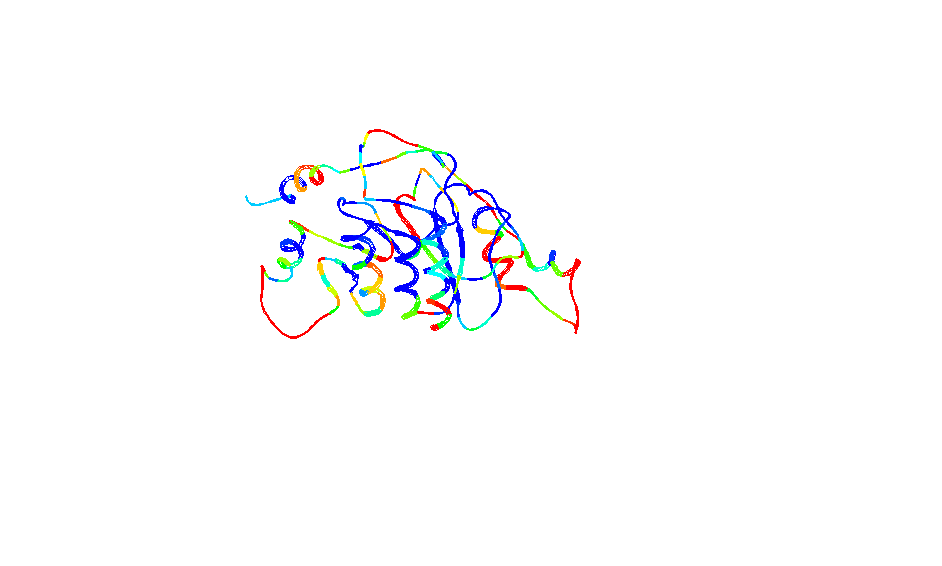

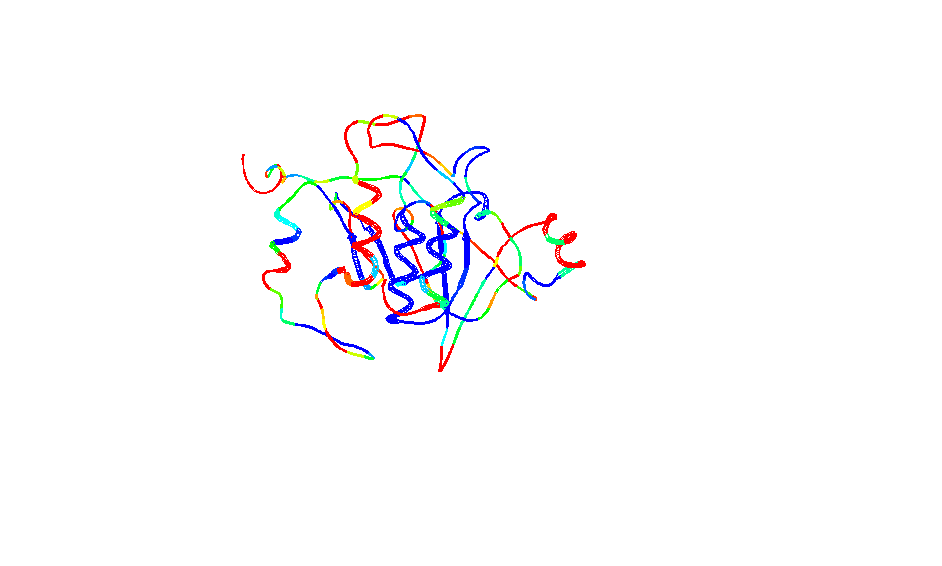


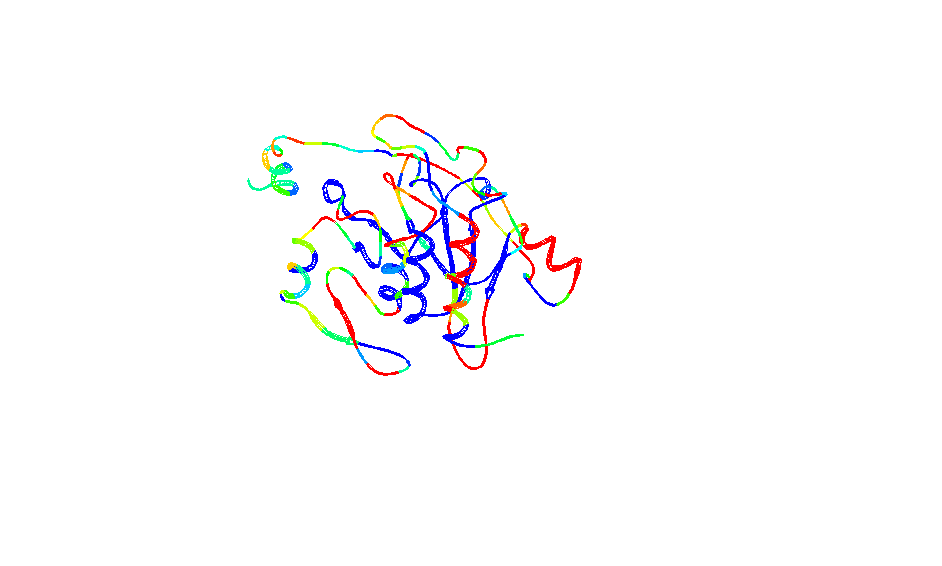

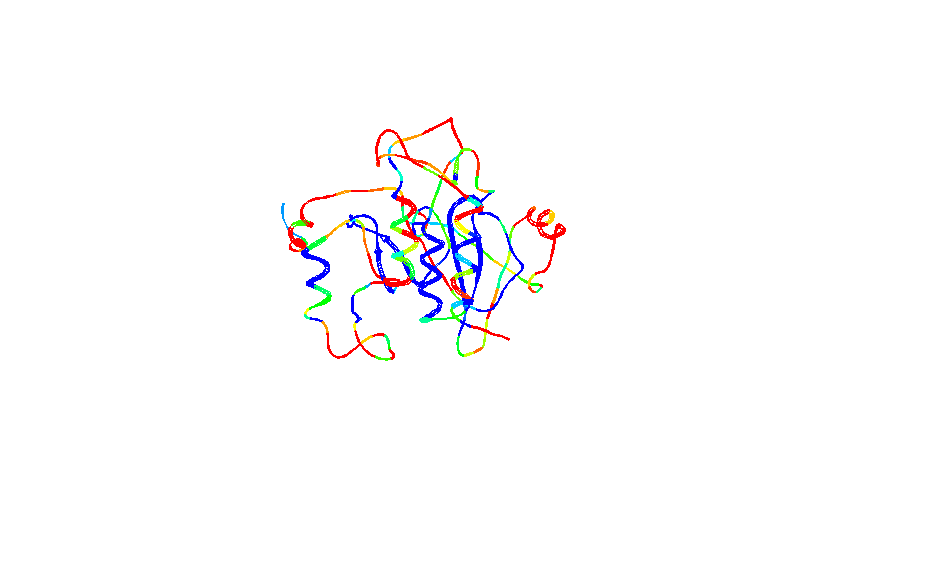

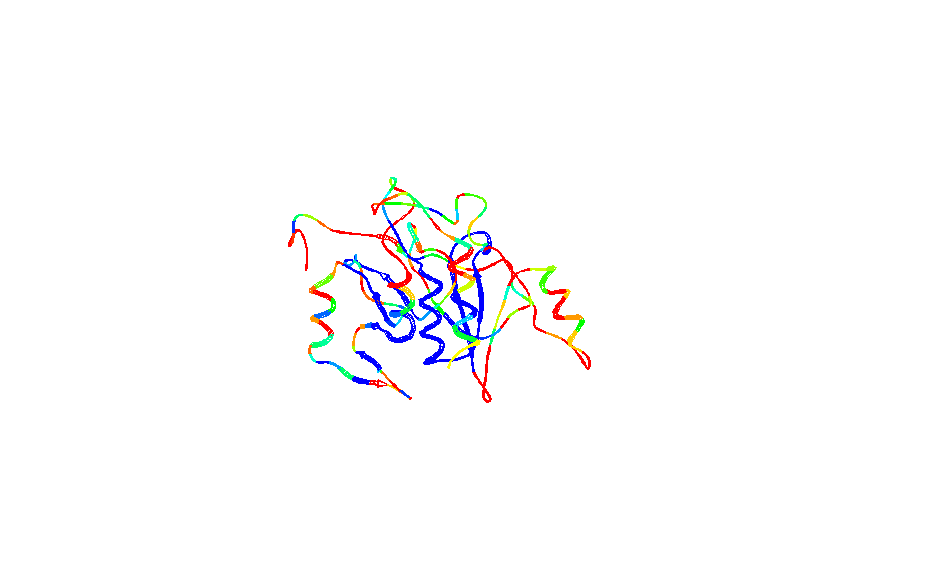

Supplement: Additional File 9 — 3-D structures of CstII (template) and modeled ST3Gals with residues color-coded based on Verify3D scores and rendered using SwissPDBViewer. The Verify3D scores were obtained using the Colorado3D server. Blue regions indicate good scores and red indicate bad scores. The rendering for CstII (top row) shows the superposition of all the 25 models generated. A representative structure from among the top three/four models is shown for ST3Gal I, II, and III (middle row, from left to right) and ST3Gal IV, V and VI (bottom row, from left to right). The average scores per residue obtained using window size 5 are as follows: 0.46, CstII; 0.27, ST3Gal I; 0.26, ST3Gal II; 0.22, ST3Gal III; 0.27, ST3Gal IV; 0.26, ST3Gal V; 0.22, ST3Gal VI. [file 1472-6807-6-9-S9.doc]
