# Supplementary material for: Fold-recognition and comparative modeling of human α2,3-sialyltransferases reveal their sequence and structural similarities to CstII from Campylobacter jejuni
Source: BMC Struct Biol. 2006 Apr 19;6:9. doi: 10.1186/1472-6807-6-9 (PMC1508147; doi:10.1186/1472-6807-6-9)
Supplement: Additional File 6 — Sequence alignments of ST3Gal I, II, III, IV, V, and VI (targets) with proteins of known 3-D structures (templates) used for modeling. The N-terminal region preceding the L-motif was modeled following the Cα-trace of the CstII C-terminus in reverse direction. The amino acid sequence of CstII in this region is italicized. 1RO7 was unanimously identified by FUGUE, FFAS03 and SAM-T02 as the template for all the ST3Gals. The names of other templates are given in the footnote to the Table in Additional file 4. The numbers at the top correspond to the sequence number of the appropriate ST3Gal. The characters above the ST3Gal sequences indicate helices and strands (nomenclature as in Figure 1); the letters H (for helix) and E (for strand) at the bottom of each block of alignment indicate the secondary structure in the template. [file 1472-6807-6-9-S6.doc]

(a) Sequence alignments used for modeling ST3Gal I structure

75 85 95 96 104 114

....|....| ....|....| ....|....| ....|....| ....|....| ....|....|

## K K’

GQRKLSAWFD ERFNQTMQPL LTAQNALLED D--------- --TYRWWLRL QREKKPNNLN **ST3Gal I**

*INKSFKGYAE SSPILIDKTY NNKEQIIFNS NLNPALEIFN AL*-------- ---------- **1RO7**

GQQDLIEWFR EKF------- ---------E A--------- --ALFEWQVQ QGDDATLSGE **1IUF**

---------- ---------- ---------- ---------- ---------- ---------- **1M2S**

---------- ---------- ---------- ---------- ---------- ---------- **1MXM**

## H H

124 132 137 147 157 167

....|....| ....|....| ....|....| ....|....| ....|....| ....|....|

## J 8 1 A 2

DTIKELFRVV PGNVD--PML EK-----RSV GCRRCAVVGN SGNLRESSYG PEIDSHDFVL **ST3Gal I**

---------- --*LSNPC*--- -*LCYLKI*--- -MKKVIIAGN GPSLKEIDYS -RLPNDFDVF **1RO7**

TIKRAAAILW HKIPE--YQD QPV------- ---------- ---------- ---------- **1IUF**

---------- ---------- ---------- ---------- ---------- ---------- **1M2S**

---------- ---------- ---------- ---------- ---------- ---------- **1MXM**

## H E H E

176 183 193 197 207

....|....| ....|....| ....|....| ....|....| ....|....| ....|....|

## 4 B 5

RMNKA-PTAG FE---ADVGT KTTHHLVYPE SFRE------ ---------- LGDNVSMILV **ST3Gal I**

RCNQFY---- FEDKY-YLGK KCKAVFYNPS LFFEQYYTLK HLIQNQEYET -----ELIMC **1RO7**

---------- ---------- ---------- ---------- ---------- ---------- **1IUF**

---------- ---------- ---------- ---------- ---------- ---------- **1M2S**

---------- ---------- ---------- ---------- ---------- ---------- **1MXM**

## E H E

212 218 227 235 245 249

....|....| ....|....| ....|....| ....|....| ....|....| ....|....|

## C 5’ 6

PFKTI----- ----DLEWVV SAITTGTIS- -HTYIPVPA- KIRVKQDKIL IYHP------ **ST3Gal I**

SN---YNQAH LENENFVKTF YD-------Y F--------- ----PDAHLG ----YDFFKQ **1RO7**

---------- ---------- ---------- ---------- ---------- ---------- **1IUF**

---------- ----SECWTA CKKVTGSGQ- ---------- ---------- ---------- **1M2S**

---------- ---------- ---------- ----VQIFST TMRTADGKII VI-------- **1MXM**

## H E E

258 268 278 286 296 306

....|....| ....|....| ....|....| ....|....| ....|....| ....|....|

## E F 7

-AFIKYVFDN WLQGHGRYPS TGILSVIFSM HVC--DEVDL YGFGADSKGN WHHYWENNPS **ST3Gal I**

LKDFNAYFKF HEIYFNQRIT SGVYMCAVAI AL-GYKEIYL SGIDFYQNGS SYAFDTKQKN **1RO7**

---------- ---------- ---------- ---------- ---------- ---------- **1IUF**

---------- ---------- ---------- ---------- ---------- ---------- **1M2S**

---------- ---------- ---------- ---------- ---------- ---------- **1MXM**

## H H E

308 312 322 332

....|....| ....|....| ....|....| ....|....|

## I

AG-------- ------AFRK TGVHDADFES NVTATLASIN  **ST3Gal I**

LLKLAPNFKN DNSHYI---- --GHSKNTDI KALEFLEKTY  **1RO7**

---------- ---------- ---------- ----------  **1IUF**

---------- ---------- ---------- ----------  **1M2S**

---------- ---------- ---------- ----------  **1MXM**

**H**

(b) Sequence alignments used for modeling ST3Gal II structure

85 95 105 110 120 130

....|....| ....|....| ....|....| ....|....| ....|....| ....|....|

## K K’ J

MGDAGASDWF DSHFDGNISP VWTRENMDLP PDVQR----- WWMMLQPQFK SHNTNEVLEK **ST3Gal II**

--*INKSFKGY AESSPILIDK TYNNKEQIIF NSNLNPALEI* ---------- ---------- **1RO7**

SGQQDLIEWF REKFGKD--- ---------- ---------- ---------- ---------- **1IUF**

---------- ---------- ---------- ---------- ---------- ---TAEQVIA **1H8B**

---------- ---------- ---------- ---------- ---------- ---------- **1M2S**

---------- ---------- ---------- ---------- ---------- ---------- **1CPT**

---------- ---------- ---------- ---------- ---------- ---------- **1ACW**

## H H

140 148 157 167 177 186

....|....| ....|....| ....|....| ....|....| ....|....| ....|....|

## 1 A 2

LFQIVPGENP YRFRDPHQ-- -CRRCAVVGN SGNLRGSGYG QDVDGHNFIM RMNQA-PTVG **ST3Gal II**

-*FNALLSNPC LCY*-----*LK I*MKKVIIAGN GPSLKEIDYS -RLPNDFDVF RCNQFY---- **1RO7**

---------- ---------- ---------- ---------- ---------- ---------- **1IUF**

SFRILASDKP Y--------- ---------- ---------- ---------- ---------- **1H8B**

---------- ---------- ---------- ---------- ---------- ---------- **1M2S**

---------- ---------- ---------- ---------- ---------- ---------- **1CPT**

---------- ---------- ---------- ---------- ---------- ---------- **1ACW**

## E H E

193 203 207 217 222

....|....| ....|....| ....|....| ....|....| ....|....| ....|....|

## 4 5

FE---QDVGS RTTHHFMYPE SAKN------ ---------- LPANVSFVLV PFKVL----- **ST3Gal II**

FEDKY-YLGK KCKAVFYNPS LFFEQYYTLK HLIQNQEYET -----ELIMC SN---YNQAH **1RO7**

---------- ---------- ---------- ---------- ---------- ---------- **1IUF**

---------- ---------- ---------- ---------- ---------- ---------- **1H8B**

---------- ---------- ---------- ---------- ---------- ---------- **1M2S**

---------- ---------- ---------- ---------- ---------- ---------- **1CPT**

---------- ---------- ---------- ---------- ---------- ---------- **1ACW**

## E H E

228 237 244 254 259 267

....|....| ....|....| ....|....| ....|....| ....|....| ....|....|

## C 5’ 6 E

----DLLWIA SALSTGQIR- ---FTYAPVK SFLRVDKEKV QIYNP----- --AFFKYIHD **ST3Gal II**

LENENFVKTF YD-------Y FPD------- --------AH LG---YDFFK QLKDFNAYFK **1RO7**

---------- ---------- ---------- ---------- ---------- ---------- **1IUF**

---------- ---------- ---------- ---------- ---------- ---------- **1H8B**

----SECWTA CKKVTGSGQ- ---------- ---------- ---------- ---------- **1M2S**

---------- ---------- ------APVK SF-------- ---------- ---------- **1CPT**

---------- ---------- ---------- AQAKCDNDKC VCE------- ---------- **1ACW**

## H E E H

277 287 295 305 315 318

....|....| ....|....| ....|....| ....|....| ....|....| ....|....|

## F 7

RWTEHHGRYP STGMLVLFFA LHVC--DEVN VYGFGADSRG NWHHYWENNR YAG------- **ST3Gal II**

FHEIYFNQRI TSGVYMCAVA IAL-GYKEIY LSGIDFYQNG SSYAFDTKQK NLLKLAPNFK **1RO7**

---------- ---------- ---------- ---------- ---------- ---------- **1IUF**

---------- ---------- ---------- ---------- ---------- ---------- **1H8B**

---------- ---------- ---------- ---------- ---------- ---------- **1M2S**

---------- ---------- ---------- ---------- ---------- ---------- **1CPT**

---------- ---------- ---------- ---------- ---------- ---------- **1ACW**

## H E

321 331 341 347

....|....| ....|....| ....|....| ....|.

## I

-------EFR KTGVHDADFE AHIIDMLAKA SKIEVY **ST3Gal II**

NDNSHYI--- ---GHSKNTD IKALEFLEKT Y----- **1RO7**

---------- ---------- ---------- ------ **1IUF**

---------- ---------- ---------- ------ **1H8B**

---------- ---------- ---------- ------ **1M2S**

---------- ---------- ---------- ------ **1CPT**

---------- ---------- ---------- ------ **1ACW**

## H

(c) Sequence alignments used for modeling ST3Gal III structure

101 111 121 130 138 142

....|....| ....|....| ....|....| ....|....| ....|....| ....|....|

## K J

SALMTAIFPR FSKPAPMFLD DSFRKWARIR -EFVPPFGIK --GQDNLIKA ILSV------ **ST3Gal III**

---*INKSFKG YAESSPILID KTYNNKEQII FNSNLNPALE IF*-------- ----*NALLSN* **1RO7**

-SEAVKFLTN ETR------- ---------- ---------- ---------- ---------- **1BL1**

---------- ---------- ---------- ---------- ----RSEWDI LLKD------ **1I7B**

---------- ---------- ---------- ---------- ---------- ---------- **1V74**

---------- ---------- ---------- ---------- ---------- ---------- **1E4Q**

## H H

152 162 172 181 189 198

....|....| ....|....| ....|....| ....|....| ....|....| ....|....|

## 8 1 A 2 3

TKEYRLTPAL DSLRCRRCII VGNGGVLANK S-LGSRIDDY D--IVVRLNS -APVKGFEKD **ST3Gal III**

*PC-LCYLKI*- ----MKKVII AGNGPSLKEI -DYSRLPNDF DVF---RCNQ F-----YFED **1RO7**

---------- ---------- ---------- ---------- ---------- ---------- **1BL1**

VQ-C------ ---------- ---------- ---------- ---------- ---------- **1I7B**

---------- ---------- ---------- ---------- ---------- ---------- **1V74**

---------- ---------- ---------- ---------- ---------- ---------- **1E4Q**

## E E H E

205 215 216 222 230 235

....|....| ....|....| ....|....| ....|....| ....|....| ....|....|

## 4 B 5

---VGSKTTL RITYPEGAMQ R--------- ----PEQYER DSLFVLAG-- -----FKWQD **ST3Gal III**

KYYLGKKCKA VFYNPSLFFE QYYTLKHLIQ NQEY-----E TELIMCSNYN QAHLE----N **1RO7**

---------- ---------- ---------- ---------- ---------- ---------- **1BL1**

---------- ---------- ---------- ---------- ---------- ---------- **1I7B**

---------- ---------- ---------- ---------- ---------- ---------F **1V74**

---------- ---------- ---------- ---------- ---------- ---------- **1E4Q**

## H E H E

245 255 257 259 269 273

....|....| ....|....| ....|....| ....|....| ....|....| ....|....|

## C 5’ 6

FKWLKYIVYK ERVSASDGFW KS-------- --------VA TRVPKEPPEI RIL--N---- **ST3Gal III**

---------- ---------- --ENFVKTFY DYF------- ------PDAH LGYDF-FKQL **1RO7**

---------- ---------- ---------- ---------- ---------- ---------- **1BL1**

---------- ---------- ---------- ---------- ---------- ---------- **1I7B**

RDAIEEHLSD KDT-VEKGTY RR-------- ---EKG---- SKVYFNPNTM NVV------- **1V74**

---------- ---------- ---------- ---------- ---------- ---------- **1E4Q**

## H E E E

283 293 299 309 316 326

....|....| ....|....| ....|....| ....|....| ....|....| ....|....|

## E F 7

PYFIQEAAFT LIGLPFNNGL MGRGNI---- PTLGSVAVTM ALHGC---DE VAVAGFGYDM **ST3Gal III**

KDFNAYFKFH EIY------- ------FNQR ITSGVYMCAV AIALGYKE-- IYLSGID--F **1RO7**

---------- ---------- ---------- ---------- ---------- ---------- **1BL1**

---------- ---------- ---------- ---------- ---------- ---------- **1I7B**

---------- ---------- ---------- ---------- ---------- ---------- **1V74**

---------- ---HPVFCPR RYK-QI---- ---------- ---------- ---------- **1E4Q**

## H H E

336 345 349 350 360 369

....|....| ....|....| ....|....| ....|....| ....|....| ....|....

## I

STPNAPLHYY E-TVRMAAIK ESWT------ ---------H NIQREKEFLR KLVKARVIT **ST3Gal III**

YQNGSSYAFD TKQKNLL--- ----KLAPNF KNDNSHYIGH SKNTDIKALE FLEKTY--- **1RO7**

---------- ---------- ---------- ---------- ---------- --------- **1BL1**

---------- ---------- ---------- ---------- ---------- --------- **1I7B**

---------- ---------- ---------- ---------- ---------- --------- **1V74**

---------- ---------- ---------- ---------- ---------- --------- **1E4Q**

## E H

(d) Sequence alignments used for modeling ST3Gal IV structure

60 70 80 90 98 102

....|....| ....|....| ....|....| ....|....| ....|....| ....|....|

## K J

ESKASKLFGN YSRDQPIFLR LEDYFWVKTP SAYELPYGTK --GSEDLLLR VLAI------ **ST3Gal IV**

---*INKSFKG YAESSPILID KTYNNKEQII FNSNLNPALE IF*-------- ----*NALLSN* **1RO7**

-SEAVKFLTN ETR------- ---------- ---------- ---------- ---------- **1BL1**

---------- ---------- ---------- ---------- ----RSEWDI LLKD------ **1I7B**

---------- ---------- ---------- ---------- ---------- ---------- **1V74**

---------- ---------- ---------- ---------- ---------- ---------- **1E4Q**

## H H

112 122 132 141 148 157

....|....| ....|....| ....|....| ....|....| ....|....| ....|....|

## 8 1 A 2

TSSSIPKNIQ SLRCRRCVVV GNGHRLRNSS -LGDAINKYD --VVIRLNN- APVAGYEGD- **ST3Gal IV**

*PCLCYLKI*-- ---MKKVIIA GNGPSLKEI- DYSRLPNDFD VF---RCNQF -----YFEDK **1RO7**

---------- ---------- ---------- ---------- ---------- ---------- **1BL1**

VQC------- ---------- ---------- ---------- ---------- ---------- **1I7B**

---------- ---------- ---------- ---------- ---------- ---------- **1V74**

---------- ---------- ---------- ---------- ---------- ---------- **1E4Q**

## E E H E E

165 175 175 182 190 195

....|....| ....|....| ....|....| ....|....| ....|....| ....|....|

## 4 B 5

--VGSKTTMR LFYPESAHFD ---------- ---PKVENNP DTLLVLVA-- -----FKAMD **ST3Gal IV**

YYLGKKCKAV FYNPSLFFEQ YYTLKHLIQN QEY------E TELIMCSNYN QAHLE----N **1RO7**

---------- ---------- ---------- ---------- ---------- ---------- **1BL1**

---------- ---------- ---------- ---------- ---------- ---------- **1I7B**

---------- ---------- ---------- ---------- ---------- ---------F **1V74**

---------- ---------- ---------- ---------- ---------- ---------- **1E4Q**

## E H E

205 215 216 219 229 233

....|....| ....|....| ....|....| ....|....| ....|....| ....|....|

## C 5’ 6

FHWIETILSD KKRVRKGFWK Q--------- -------PPL IWDVNPKQIR IL--N----P **ST3Gal IV**

---------- ---------- -ENFVKTFYD YF-------- -----PDAHL GYDF-FKQLK **1RO7**

---------- ---------- ---------- ---------- ---------- ---------- **1BL1**

---------- ---------- ---------- ---------- ---------- ---------- **1I7B**

RDAIEEHLSD KDTVEKGTYR R--------- --EKGSK--- -VYFNPNTMN VV-------- **1V74**

---------- ---------- ---------- ---------- ---------- ---------- **1E4Q**

## H E E

243 253 259 269 276 286

....|....| ....|....| ....|....| ....|....| ....|....| ....|....|

## E F 7

FFMEIAADKL LSLPMQQPRK IKQK----PT TGLLAITLAL HLC---DLVH IAGFGYPDAY **ST3Gal IV**

DFNAYFKFHE IY-------- ----FNQRIT SGVYMCAVAI ALGYKE--IY LSGID---FY **1RO7**

---------- ---------- ---------- ---------- ---------- ---------- **1BL1**

---------- ---------- ---------- ---------- ---------- ---------- **1I7B**

---------- ---------- ---------- ---------- ---------- ---------- **1V74**

---------- --HPVFCPRR YKQI------ ---------- ---------- ---------- **1E4Q**

## H H E

296 305 307 310 320 329

....|....| ....|....| ....|....| ....|....| ....|....| ....|....

## I

NKKQTIHYYE -QITLKSMAG SG-------- -------HNV SQEALAIKRM LEMGAIKNL **ST3Gal IV**

QNGSSYAFDT KQKNLL---- --KLAPNFKN DNSHYIGHSK NTDIKALEFL EKTY----- **1RO7**

---------- ---------- ---------- ---------- ---------- --------- **1BL1**

---------- ---------- ---------- ---------- ---------- --------- **1I7B**

---------- ---------- ---------- ---------- ---------- --------- **1V74**

---------- ---------- ---------- ---------- ---------- --------- **1E4Q**

**E H**

(e) Sequence alignments used for modeling ST3Gal V structure

79 89 99 109 117 121

....|....| ....|....| ....|....| ....|....| ....|....| ....|....|

## K J

KTSMALLFEH RYSVDLLPFV QKAPKDSEAE SKYDPPFGFR --KFSSKVQT LLEL------ **ST3Gal V**

---*INKSFKG YAESSPILID KTYNNKEQII FNSNLNPALE IF*-------- ----*NALLSN* **1RO7**

-SEAVKFLTN ETR------- ---------- ---------- ---------- ---------- **1BL1**

---------- ---------- ---------- ---------- ----RSEWDI LLKD------ **1I7B**

---------- ---------- ---------- ---------- ---------- ---------- **1V74**

---------- ---------- ---------- ---------- ---------- ---------- **1E4Q**

## H H

131 141 151 160 168 177

....|....| ....|....| ....|....| ....|....| ....|....| ....|....|

## 8 1 A 2 3

LPEHDLPEHL KAKTCRRCVV IGSGGILHGL E-LGHTLNQF D--VVIRLNS -APVEGYSEH **ST3Gal V**

*PC-LCYLKI*- ----MKKVII AGNGPSLKEI -DYSRLPNDF DVF---RCNQ F-----YFED **1RO7**

---------- ---------- ---------- ---------- ---------- ---------- **1BL1**

VQ-C------ ---------- ---------- ---------- ---------- ---------- **1I7B**

---------- ---------- ---------- ---------- ---------- ---------- **1V74**

---------- ---------- ---------- ---------- ---------- ---------- **1E4Q**

## E E H E

184 194 195 200 209 213

....|....| ....|....| ....|....| ....|....| ....|....| ....|....|

## 4 5

---VGNKTTI RMTYPEGAPL S--------- ----D-LEYY SNDLFVAVL- ------FKSV **ST3Gal V**

KYYLGKKCKA VFYNPSLFFE QYYTLKHLIQ NQEY------ ETELIMCSNY NQAHLE---- **1RO7**

---------- ---------- ---------- ---------- ---------- ---------- **1BL1**

---------- ---------- ---------- ---------- ---------- ---------- **1I7B**

---------- ---------- ---------- ---------- ---------- ---------- **1V74**

---------- ---------- ---------- ---------- ---------- ---------- **1E4Q**

## E E H E

223 233 238 238 247 254

....|....| ....|....| ....|....| ....|....| ....|....| ....|....|

## C 5’ 6

DFNWLQAMVK KETLPFWVRL FFWKQ----- ---------- -VAEKIPLQP KHFRIL--N- **ST3Gal V**

N--------- ---------- -----ENFVK TFYDYF---- ---------P DAHLGYDF-F **1RO7**

---------- ---------- ---------- ---------- ---------- ---------- **1BL1**

---------- ---------- ---------- ---------- ---------- ---------- **1I7B**

FRDAIEEHLS DKDT---VEK GTYRR----- ------EKGS K----VYFNP NTMNVV---- **1V74**

---------- ---------- ---------- ---------- ---------- ---------- **1E4Q**

## H E E

261 271 281 287 297 304

....|....| ....|....| ....|....| ....|....| ....|....| ....|....|

## E F 7

---PVIIKET AFDILQYSEP QSRFWGRDKN V----PTIGV IAVVLATHLC ---DEVSLAG **ST3Gal V**

KQLKDFNAYF KFHEIY---- ---------- -FNQRITSGV YMCAVAIALG YKE--IYLSG **1RO7**

---------- ---------- ---------- ---------- ---------- ---------- **1BL1**

---------- ---------- ---------- ---------- ---------- ---------- **1I7B**

---------- ---------- ---------- ---------- ---------- ---------- **1V74**

---------- ------HPVF CPRRY--KQI ---------- ---------- ---------- **1E4Q**

## H H E

313 322 332 332 337 347

....|....| ....|....| ....|....| ....|....| ....|....| ....|....|

## I

FGY-DLNQPR TPLHYFD-SQ CMAAMNFQTM ---------- -----HNVTT ETKFLLKLVK **ST3Gal V**

ID---FYQNG SSYAFDTKQK NLL------- KLAPNFKNDN SHYIGHSKNT DIKALEFLEK **1RO7**

---------- ---------- ---------- ---------- ---------- ---------- **1BL1**

---------- ---------- ---------- ---------- ---------- ---------- **1I7B**

---------- ---------- ---------- ---------- ---------- ---------- **1V74**

---------- ---------- ---------- ---------- ---------- ---------- **1E4Q**

## E H

354

....|..

EGVVKDL **ST3Gal V**

TY----- **1RO7**

------- **1BL1**

------- **1I7B**

------- **1V74**

**------- 1E4Q**

(f) Sequence alignments used for modeling ST3Gal VI structure

59 68 78 83 91 100

....|....| ....|....| ....|....| ....|....| ....|....| ....|....|

## J

FASLL-RFHQ FHP-FLCAAD FRKIASLYGS DKFDL----- --PYGMRTSA EYFRLALSK- **ST3Gal VI**

*INKSFKGYAE SSPILIDKTY NNKEQI---I FNSNLNPALE IF*-------- ---------*N* **1RO7**

---------- ---------- ---------- ---------- ----GKKEAY DTLIKDLKK- **1DDF**

---------- ---------- ---------- ---------- ---------- ---------- **1V74**

---------- ---------- ---------- ---------- ---------- ---------- **1YUA**

## H H

105 115 125 134 142 151

....|....| ....|....| ....|....| ....|....| ....|....| ....|....|

## 8 1 A 2 3

-----LQSCD LFDEFDNIPC KKCVVVGNGG VLKNKT-LGE KIDSYD--VI IRMNN-GPVL **ST3Gal VI**

*ALLSNPCLCY LKI*------M KKVIIAGNGP SLKEI-DYSR LPNDFDVF-- -RCNQF---- **1RO7**

-----ANL-- ---------- ---------- ---------- ---------- ---------- **1DDF**

---------- ---------- ---------- ---------- ---------- ---------- **1V74**

---------- ---------- ---------- ---------- ---------- ---------- **1YUA**

## E E H E

158 168 174 175 185 188

....|....| ....|....| ....|....| ....|....| ....|....| ....|....|

## 4 B 5

GHEEE---VG RRTTFRLFYP ESVFSD---- ---------P IHNDPNTTVI LTA------- **ST3Gal VI**

-YFEDKYYLG KKCKAVFYNP SLFFEQYYTL KHLIQNQEY- ----ETELIM CSNYNQAHLE **1RO7**

---------- ---------- ---------- ---------- ---------- ---------- **1DDF**

---------- ---------- ---------- ---------- ---------- ---------- **1V74**

---------- ---------- ---------- ---------- ---------- ---------- **1YUA**

## E E H E

198 208 214 214 222 230

....|....| ....|....| ....|....| ....|....| ....|....| ....|....|

## C 5’ 6

FKPHDLRWLL ELLMGDKINT NGFWKK---- ---------- --PALNLIYK PYQIRIL--D **ST3Gal VI**

----N----- ---------- ------ENFV KTFYDYF--- ---------- PDAHLGYDF- **1RO7**

---------- ---------- ---------- ---------- ---------- ---------- **1DDF**

----FRDAIE EHLSDKDTVE KGTYRR---- -------EKG SK----VYFN PNTMNVV--- **1V74**

---------- ---------- ---------- ---------- ---------- ---------- **1YUA**

## H E E E

236 243 252 262 271 279

....|....| ....|....| ....|....| ....|....| ....|....| ....|....|

## E F 7

----PFIIRT AAYELLH--- -FPKVFPKNQ KPKHPTTGII AITLAFYIC- --HEVHLAGF **ST3Gal VI**

FKQLKDFNAY FKFHEIYFNQ R--------- ----ITSGVY MCAVAIALGY KE--IYLSGI **1RO7**

---------- ---------- ---------- ---------- ---------- ---------- **1DDF**

---------- ---------- ---------- ---------- ---------- ---------- **1V74**

---------- ---------- ---NTFPKSR ETRAPLVE-- ---------- ---------- **1YUA**

## H H E

289 298 306 306 313 323

....|....| ....|....| ....|....| ....|....| ....|....| ....|....|

## I

KYNFSDLKSP LHYYG-NATM SLMNKNAY-- ---------- ---HNVTAEQ LFLKDIIEKN **ST3Gal VI**

D--FYQNGSS YAFDTKQKNL L-------KL APNFKNDNSH YIGHSKNTDI KALEFLEKTY **1RO7**

---------- ---------- ---------- ---------- ---------- ---------- **1DDF**

---------- ---------- ---------- ---------- ---------- ---------- **1V74**

---------- ---------- ---------- ---------- ---------- ---------- **1YUA**

**E H**
