# Supplementary material for: Fold-recognition and comparative modeling of human α2,3-sialyltransferases reveal their sequence and structural similarities to CstII from Campylobacter jejuni
Source: BMC Struct Biol. 2006 Apr 19;6:9. doi: 10.1186/1472-6807-6-9 (PMC1508147; doi:10.1186/1472-6807-6-9)
Supplement: Additional File 7 — Stereo chemical qualities of the generated models. The values are for the top three models of ST3Gals except ST3Gal VI, for which the values are reported for the top four models. The average score per residue with different window sizes were calculated using the Colorado3D server. The range of scores obtained for the 25 models obtained using Modeller, Procheck and Verify3D are reported for CstII. The modeling was done by aligning the CstII sequence with its own structure (PDB id 1RO7). [file 1472-6807-6-9-S7.doc]

| **Protein** | **Models created** | **Best model**  **No.** | **Modeller energy** | **Ramachandran Plot: % residues in regions** | | | | **Bad contacts /100 residues** | **Verify 3D** | | | |
| --- | --- | --- | --- | --- | --- | --- | --- | --- | --- | --- | --- | --- |
|  |  |  |  | **Most favored** | **Allowed** | **Generous** | **Disallowed** |  | **Min** | **Max** | **Average score / residue with window size** | |
|  |  |  |  |  |  |  |  |  |  |  | **21** | **5** |
| Cst II^*^ | 25 | 12 | 1334 to 2101 | 89.1 to 91.2 | 7.6 to 10.1 | 0.4 to 1.3 | 0.0 to 0.8 | -0.3 to 0.0 inside | -0.09 to 0.15 | 0.77 to 0.91 | 0.42 to 0.51 | 0.42 to 0.51 |
| ST3Gal I | 25 | 3 | 1672 | 86 | 11.1 | 2.1 | 0.9 | -0.3-inside | -0.02 | 0.7 | 0.26 | 0.27 |
|  |  | 9 | 1903 | 88.1 | 9.4 | 0.9 | 1.7 | -0.1-inside | -0.09 | 0.66 | 0.24 | 0.24 |
|  |  | 17 | 1740 | 83 | 14.9 | 0.4 | 1.7 | 0.3- inside | -0.09 | 0.75 | 0.24 | 0.25 |
| ST3Gal II | 25 | 3 | 1538 | 89.4 | 9.7 | 0.8 | 0.0 | -0.2-inside | -0.09 | 0.74 | 0.26 | 0.26 |
|  |  | 8 | 1849 | 86 | 11.9 | 1.7 | 0.4 | -0.2-inside | -0.12 | 0.74 | 0.26 | 0.26 |
|  |  | 17 | 1954 | 87.7 | 10.2 | 0.8 | 1.3 | 0.2-inside | -0.12 | 0.8 | 0.25 | 0.25 |
| ST3Gal III | 25 | 25 | 1662 | 87.5 | 11.7 | 0.8 | 0.0 | -0.1-inside | -0.07 | 0.75 | 0.23 | 0.22 |
|  |  | 14 | 1780 | 87.1 | 10.4 | 1.7 | 0.8 | -0.2-inside | -0.1 | 0.72 | 0.22 | 0.21 |
|  |  | 7 | 1688 | 87.5 | 10.8 | 1.3 | 0.4 | -0.1-inside | -0.11 | 0.73 | 0.21 | 0.21 |
| ST3Gal IV | 25 | 12 | 1701 | 88.2 | 10.2 | 1.6 | 0.0 | -0.2-inside | -0.11 | 0.71 | 0.18 | 0.19 |
|  |  | 13 | 1688 | 87.8 | 7.8 | 3.3 | 1.2 | 0.2-inside | -0.1 | 0.77 | 0.24 | 0.25 |
|  |  | 20 | 1768 | 83.7 | 12.7 | 2.0 | 1.6 | 0.1-inside | -0.11 | 0.82 | 0.19 | 0.19 |
| ST3Gal V | 25 | 6 | 1644 | 86.9 | 11.1 | 2.0 | 0.0 | 0.0-inside | -0.16 | 0.84 | 0.20 | 0.21 |
|  |  | 13 | 1733 | 87.7 | 10.3 | 1.2 | 0.8 | -0.3-inside | -0.1 | 0.69 | 0.20 | 0.20 |
|  |  | 22 | 1909 | 83.3 | 12.3 | 2.4 | 2.0 | -1.0-inside | -0.09 | 0.69 | 0.23 | 0.23 |
| ST3Gal VI | 25 | 7 | 1599 | 86.7 | 10.8 | 2.5 | 0.0 | -0.3-inside | -0.18 | 0.67 | 0.16 | 0.16 |
|  |  | 13 | 1550 | 84.2 | 12.9 | 2.1 | 0.8 | -0.2-inside | -0.08 | 0.75 | 0.22 | 0.22 |
|  |  | 22 | 1643 | 88.0 | 10.0 | 2.1 | 0.0 | -0.1-inside | -0.17 | 0.8 | 0.22 | 0.22 |
|  |  | 24 | 1690 | 88.4 | 10 | 0.4 | 1.2 | -0.2-inside | -0.18 | 0.78 | 0.14 | 0.14 |
