# Supplementary material for: Fold-recognition and comparative modeling of human α2,3-sialyltransferases reveal their sequence and structural similarities to CstII from Campylobacter jejuni
Source: BMC Struct Biol. 2006 Apr 19;6:9. doi: 10.1186/1472-6807-6-9 (PMC1508147; doi:10.1186/1472-6807-6-9)
Supplement: Additional File 10 — Structural comparisons between the modeled ST3Gals and CstII. The SSM [57] and DALI [56] servers were used for structure comparison. Both the servers identify CstII as the top hit. RMSD represents root mean square deviation calculated between Cα-atoms of matched residues at best 3D superposition of the query and target structures. Nalign represents the number of matched residues between the query and target. Qscore is a quality function of Cα-alignment. It's a combined parameter for Nalign and RMSD. The identical structures have a Qscore of 1. Zscore is a statistical significance score for best domain-domain alignment. [file 1472-6807-6-9-S10.doc]

| Query Protein | **SSM** | | | | **DALI** | | | |
| --- | --- | --- | --- | --- | --- | --- | --- | --- |
|  | Target Protein | RMSD | Nalign | Qscore | Target Protein | RMSD | Nalign | Zscore |
| ST3Gal I | 1RO7:C | 1.38 | 157 | 0.31 | 1RO7:A | 1.7 | 168 | 16.6 |
| ST3Gal II | 1RO7:C | 1.66 | 161 | 0.29 | 1RO7:A | 1.9 | 170 | 16.6 |
| ST3Gal III | 1RO7:C | 1.96 | 153 | 0.24 | 1RO7:A | 2.4 | 174 | 15.7 |
| ST3Gal IV | 1RO8:B | 1.98 | 137 | 0.21 | 1RO7:A | 2.8 | 172 | 14.3 |
| ST3Gal V | 1RO7:C | 1.79 | 154 | 0.25 | 1RO7:A | 2.8 | 175 | 14.7 |
| ST3Gal VI | 1RO7:C | 2.62 | 114 | 0.11 | 1RO7:A | 2.7 | 173 | 14.5 |
