# Supplementary material for: Fold-recognition and comparative modeling of human α2,3-sialyltransferases reveal their sequence and structural similarities to CstII from Campylobacter jejuni
Source: BMC Struct Biol. 2006 Apr 19;6:9. doi: 10.1186/1472-6807-6-9 (PMC1508147; doi:10.1186/1472-6807-6-9)
Supplement: Additional File 11 — Multiple sequence alignment of experimentally characterized mammalian α2,3-SiaTs and SiaTs from GT42 family. Only the experimentally characterized SiaT sequences have been taken from both the families. The sequence alignment was generated by first aligning mammalian SiaTs with CstII using FUGUE; this was used as guide to merge the multiple sequence alignments of mammalian ST3Gals and GT42 family members. The L-, S- and VS-motif regions have been marked in the alignment. The regions of protein marked in red were used to generate the sequence logos (Figure 7). The sequences used are: bifunctional α2,3/8-sialyltransferase, CstII (1RO7); α2,3-sialyltransferase, CstI (AAF13495); α2,3-sialyltransferase, CstII (AAF34137); bifunctional α2,3/8-sialyltransferase, CstII (AAL06004); α2,3-sialyltransferase, CstI (AAF13495); α2,3-sialyltransferase, CstII (AAF34137); bifunctional α2,3/8-sialyltransferase, CstII (AAL06004), α2,3-sialyltransferase, CstIII (AAK73183); α2,3/8-sialyltransferase, CstII (AAF31771); all these proteins are from Campylobacter jejuni. The experimentally characterized mammalian α2,3 sialyltransferases are taken from [11]. ST3Gal I (Q11201, human; P54751, mouse; Q11200, chick; Q02745, pig), ST3Gal II (Q16842, human; NP_835149, mouse), ST3Gal III (Q11203, human; P97325, mouse and Q02734, rat), ST3Gal IV (Q11206, human; NP_033204, mouse), ST3Gal V (Q9UNP4, human; O88829, mouse) and ST3Gal VI (Q9Y274, human). The H. influenzae sequences in family GT42 have not been used in the multiple sequence alignment, and hence to generate sequence logos, because all these SiaTs are computationally annotated sequences. [file 1472-6807-6-9-S11.doc]

10 20 30 40 50 60

....|....| ....|....| ....|....| ....|....| ....|....| ....|....|

---------------------L motif ------------------

---------- ------kkVI IAGNGpsLke IdysrLPndf d-VFRCNQFY FEd-kYYLgk **1RO7A**

MTRTRMENEL IVSKNMQNII IAGNGPSLKN INYKRLPREY D-VFRCNQFY FED-KYYLGK **AAF13495**

---------- -----MKKVI IAGNGPSLKE IDYSRLPNDF D-VFRCNQFY FED-KYYLGK **AAF34137**

---------- -----MKKVI ISGNGPSLKE IDYSRLPNDF D-VFRCNQFY FED-KYYLGK **AAL06004**

MTRTRMENEL IVSKNMQNII IAGNGPSLKN INYKRLPREY D-VFRCNQFY FED-KYYLGK **AAF13495**

---------- -----MKKVI IAGNGPSLKE IDYSRLPNDF D-VFRCNQFY FED-KYYLGK **AAF34137**

---------- -----MKKVI ISGNGPSLKE IDYSRLPNDF D-VFRCNQFY FED-KYYLGK **AAL06004**

---------- --MSMNINAL VCGNGPSLKN IDYKRLPKQF D-VFRCNQFY FED-RYFVGK **AAK73183**

---------- -----MKKVI IAGNGPSLKE IDYSRLPNDF D-VFRCNQFY FED-KYYLGK **AAF31771**

---------- -----CRRCA VVGNSGNLRE SSYGPEIDSH DFVLRMNKAP TAGFEADVGT **Q11201**

---------- -----CRRCA VVGNSGNLKD SSYGPEIDSH DFVLRMNKAP TVGFEADVGS **P54751**

---------- -----CRRCA VVGNSGNLRQ SQYGQDIDSH DFVLRMNRAP TIGYESDVGS **Q11200**

---------- -----CRRCA VVGNSGNLKE SYYGPQIDSH DFVLRMNKAP TEGFEADVGS **Q02745**

---------- -----CRRCA VVGNSGNLRG SGYGQDVDGH NFIMRMNQAP TVGFEQDVGS **Q16842**

---------- -----CRRCA VVGNSGNLRG SGYGQEVDSH NFIMRMNQAP TVGFEKDVGS **NP_835149**

---------- -----CRRCV VVGNGHRLRN SSLGDAINKY DVVIRLNNAP VAGYEGDVGS **Q11203**

---------- -----CRRCV VVGNGHRLRN SSLGGVINKY DVVIRLNNAP VAGYEGDVGS **P97325**

---------- -----CRRCI IVGNGGVLAN KSLGSRIDDY DIVIRLNSAP VKGFEKDVGS **Q02734**

---------- -----CRRCI IVGNGGVLAN KSLGSRIDDY DIVVRLNSAP VKGFEKDVGS **Q11206**

---------- -----CRRCI IVGNGGVLAN KSLGSRIDDY DIVIRLNSAP VKGFERDVGS **NP_033204**

---------- -----CRRCV VIGSGGILHG LELGHTLNQF DVVIRLNSAP VEGYSEHVGN **Q9UNP4**

---------- -----CKRCV VVGNGGILHG LELGHALNQF DVVIRLNSAP VEGYSEHVGN **O88829**

---------- -----CKKCV VVGNGGVLKN KTLGEKIDSY DVIIRMNNGP VLGHEEEVGR **Q9Y274**

70 80 90 100 110 120

....|....| ....|....| ....|....| ....|....| ....|....| ....|....|

kCkAVFYnps lFfeQyyTLk hLiqnqeYeT elI-CSNynq ahlenen--- ------Fvkt **1RO7A**

KIKAVFFNPG VFLQQYHTAK QLILKNEYEI KNIFCSTFNL PFIESND--- ------FLHQ **AAF13495**

KCKAVFYTPN FFFEQYYTLK HLIQNQEYET ELIMCSNYNQ AHLENEN--- ------FVKT **AAF34137**

KCKAVFYNPS LFFEQYYTLK HLIQNQEYEI ELIMCSNYNQ AHLENEN--- ------FVKT **AAL06004**

KIKAVFFNPG VFLQQYHTAK QLILKNEYEI KNIFCSTFNL PFIESND--- ------FLHQ **AAF13495**

KCKAVFYTPN FFFEQYYTLK HLIQNQEYET ELIMCSNYNQ AHLENEN--- ------FVKT **AAF34137**

KCKAVFYNPS LFFEQYYTLK HLIQNQEYEI ELIMCSNYNQ AHLENEN--- ------FVKT **AAL06004**

DVKYVFFNPF VFFEQYYTSK KLIQNEEYNI ENIVCSTINL EYIDGFQ--- ------FVDN **AAK73183**

KCKAVFYNPI LFFEQYYTLK HLIQNQEYET ELIMCSNYNQ AHLENEN--- ------FVKT **AAF31771**

KTTHHLVYPE SFRELGD--- --NVSMILVP FKT-IDLEWV VSAITTGTIS HT-----YIP **Q11201**

RTTHHLVYPE SFRELGE--- --NVNMVLVP FKT-TDLQWV ISATTTGTIT HT-----YVP **P54751**

KTTHHFVYPE SYKELAE--- --NVSMIVIP FKT-LDLRWI VTALTTGTIN FT-----YVP **Q11200**

KTTHHFVYPE SFRELAQ--- --EVSMILVP FKT-TDLEWV ISATTTGRIS HT-----YVP **Q02745**

RTTHHFMYPE SAKNLPA--- --NVSFVLVP FKV-LDLLWI ASALSTGQIR FT-----YAP **Q16842**

RTTHHFMYPE SAKNLPA--- --NVSFVLVP FKA-LDLMWI ASALSTGQIR FT-----YAP **NP_835149**

KTTMRLFYPE SAHFDPKVEN NPDTLLVLVA FKA-MDFHWI ETILSDKKR- --VRKGFWKQ **Q11203**

KTTIRLFYPE SAHFDPKIEN NPDTLLVLVA FKA-MDFHWI ETILSDKKR- --VRKGFWKQ **P97325**

KTTLRITYPE GAMQRPE-QY ERDSLFVLAG FKW-QDFKWL KYIVYKERVS --ASDGFWKS **Q02734**

KTTLRITYPE GAMQRPE-QY ERDSLFVLAG FKW-QDFKWL KYIVYKERVS --ASDGFWKS **Q11206**

KTTLRITYPE GAMQRPE-QY ERDSLFVLAG FKW-QDFKWL KYIVYKERVS --ASDGFWKS **NP_033204**

KTTIRMTYPE GAPLSD-LEY YSNDLFVAVL FKS-VDFNWL QAMVKKETLP FWVRLFFWKQ **Q9UNP4**

KTTIRMTYPE GAPLSD-VEY YANDLFVTVL FKS-VDFKWL QAMVKNESLP FWVRLFFWKQ **O88829**

RTTFRLFYPE SVFSDP-IHN DPNTTVILTA FKP-HDLRWL LELLMGDKI- --NTNGFWKK **Q9Y274**

130 140 150 160 170 180

....|....| ....|....| ....|....| ....|....| ....|....| ....|....|

--------S motif--

FydyFpdAhl GydfFkq--- ------lkdF nayFkfhEiy fnqrITSGVY MCAVAIAlgY **1RO7A**

FYNFFPDAKL GYEVIEN--- ------LKEF YAYIKYNEIY FNKRITSGVY MCAIAIALGY **AAF13495**

FYDYFPDAHL GYDFFKQ--- ------LKEF NAYFKFHEIY FNQRITSGVY MCAVAIALGY **AAF34137**

FYDYFPDAHL GYDFFKQ--- ------LKEF NAYFKFHEIY FNQRITSGVY MCTVAIALGY **AAL06004**

FYNFFPDAKL GYEVIEN--- ------LKEF YAYIKYNEIY FNKRITSGVY MCAIAIALGY **AAF13495**

FYDYFPDAHL GYDFFKQ--- ------LKEF NAYFKFHEIY FNQRITSGVY MCAVAIALGY **AAF34137**

FYDYFPDAHL GYDFFKQ--- ------LKEF NAYFKFHEIY FNQRITSGVY MCTVAIALGY **AAL06004**

FELYFSDAFL GHEIIKK--- ------LKDF FAYIKYNEIY NRQRITSGVY MCATAVALGY **AAK73183**

FYDYFPDAHL GYDFFKQ--- ------LKDF NAYFKFHEIY FNQRITSGVY MCAVAIALGY **AAF31771**

VPAKIRVKQD KILIYHPAFI KYVFDNW--- -------LQG HGRYPSTGIL SVIFSMHV-- **Q11201**

VPPKIKVKQE KILIYHPAFI KYVFDNW--- -------LQG HGRYPSTGIL SIIFSIHI-- **P54751**

VPRKIKVRKE KVLIYNPSFI KYVYENW--- -------LQN HGRYPSTGLL SVIFALHV-- **Q11200**

VPAKIKVKKE KILIYHPAFI KYVFDRW--- -------LQG HGRYPSTGIL SVIFSLHI-- **Q02745**

VKSFLRVDKE KVQIYNPAFF KYIHDRW--- -------TEH HGRYPSTGML VLFFALHV-- **Q16842**

VKSFLRVDKE KVQIYNPAFF KYIHDRW--- -------TEH HGRYPSTGML VLFFALHV-- **NP_835149**

PPLIWDVNPK QIRILNPFFM EIAADKLLSL PMQQPR--KI KQK-PTTGLL AITLALHL-- **Q11203**

PPLIWDVNPK QVRILNPFFM EIAADKLLSL PIQQPR--KI KQK-PTTGLL AITLALHL-- **P97325**

VATRVPKEPP EIRILNPYFI QEAAFTLIGL PFNNG--LMG RGNIPTLGSV AVTMALDG-- **Q02734**

VATRVPKEPP EIRILNPYFI QEAAFTLIGL PFNNG--LMG RGNIPTLGSV AVTMALHG-- **Q11206**

VATRVPKEPP EIRILNPYFI QEAAFTLIGL PFNNG--LMG RGNIPTLGSV AVTMALHG-- **NP_033204**

VAEKIPLQPK HFRILNPVII KETAFDILQY SEPQSRFWGR DKNVPTIGVI AVVLATHL-- **Q9UNP4**

VAEKVPLQPK HFRILNPVII KETAFDILQY SEPQSRFWGH DKNIPTIGVI AVVLATHL-- **O88829**

PALNLIYKPY QIRILDPFII RTAAYELLHF PKVFPK--NQ KPKHPTTGII AITLAFYI-- **Q9Y274**

190 200 210 220 230 240

....|....| ....|....| ....|....| ....|....| ....|....| ....|....|

--------- VS motif

-keIYLSGID FYqngss-YA fdtkqknLlk lapnFkndnS hyiGHskntD IkALefLekt **1RO7A**

-KTIYLCGID FYEGDVI-YP FEAMSTNIKT IFPGIKD-FK PSNCHSKEYD IEALKLLKSI **AAF13495**

-KEIYLSGID FYQNGSS-YA FDTKQENLLK LAPDFKNDRS HYIGHSKNTD IKALEFLEKT **AAF34137**

-KEIYLSGID FYDNGGG-YA FDTKQKNLLK LAPNFKNDNS HYIGHSKNTD IKALEFLEKT **AAL06004**

-KTIYLCGID FYEGDVI-YP FEAMSTNIKT IFPGIKD-FK PSNCHSKEYD IEALKLLKSI **AAF13495**

-KEIYLSGID FYQNGSS-YA FDTKQENLLK LAPDFKNDRS HYIGHSKNTD IKALEFLEKT **AAF34137**

-KEIYLSGID FYDNGGG-YA FDTKQKNLLK LAPNFKNDNS HYIGHSKNTD IKALEFLEKT **AAL06004**

-KSIYISGID FYQDTNNLYA FDNNKKNLLN KCTGFKNQKF KFINHSMACD LQALDYLMKR **AAK73183**

-KEIYLSGID FYQNGSS-YA FDTKQKNLLK LAPNFKNDNS HYIGHSKNTD IKALEFLEKT **AAF31771**

CDEVDLYGFG A-D------- -SKGNWHHYW ENNPSAGAFR KTGVHDADFE SNVTATLASI **Q11201**

CDEVDLYGFG A-D------- -SKGNWHHYW ENNPSAGAFR KTGVHDGDFE YNITTTLAAI **P54751**

CDEVNVYGFG A-D------- -SKGHWHHYW ENNASAGAFR QTGVHDGDFE FNVTLTLASI **Q11200**

CDEVDLYGFG A-D------- -SKGNWHHYW ENNPSAGAFR KTGVHDGDFE SNVTTILASI **Q02745**

CDEVNVYGFG A-D------- -SRGNWHHYW ENNRYAGEFR KTGVHDADFE AHIIDMLAKA **Q16842**

CDEVNVYGFG A-D------- -SRGNWHHYW ENNRYAGEFR KTGVHDADFE AHIIDMLAKA **NP_835149**

CDLVHIAGFG YPD------A YNKKQTIHYY EQITLK-SMA GSG-HNVSQE ALAIKRMLEM **Q11203**

CDLVHIAGFG YPD------A SNKKQTIHYY EQITLK-SMA GSG-HNVSQE AIAIKRMLEM **P97325**

CDEVAVAGFG Y-D------M NTPNAPLHYY ETVRMA-AIK ESWTHNIQRE KEFLRKLVKA **Q02734**

CDEVAVAGFG Y-D------M STPNAPLHYY ETVRMA-AIK ESWTHNIQRE KEFLRKLVKA **Q11206**

CDEVAVAGFG Y-D------M NTPNAPLHYY ETVRMA-AIK ESWTHSIQRE KEFLRKLVKA **NP_033204**

CDEVSLAGFG Y-D------L NQPRTPLHYF DSQCMA-AMN FQTMHNVTTE TKFLLKLVKE **Q9UNP4**

CDEVSLAGFG Y-D------L SQPRTPLHYF DSQCMG-AMH WQVMHNVTTE TKFLLKLLKE **O88829**

CHEVHLAGFK Y-N------F SDLKSPLHYY GNATMS-LMN KNAYHNVTAE QLFLKDIIEK **Q9Y274**

250 260 270 280 290 300

....|....| ....|....| ....|....| ....|....| ....|....| ....|....|

ykIkLyCLCp nSlLanfiel Apnlnsnfii qeKnnytkdI liPsseaygk FskNi----- **1RO7A**

YKVNIYALCD DSILANHFPL SININNNFTL ENKHNNSIND ILLTDNTPGV SFYKNQLKAD **AAF13495**

YKIKLYCLCP NSLLANFIEL APNLNSNFII QEK-NNYTKD ILIPSSEAYG KFSKNINFKK **AAF34137**

YEIKLYCLCP NSLLANFIEL APNLNSNFII QEK-NNYTKD ILIPSSEAYG KFTKNINFKK **AAL06004**

YKVNIYALCD DSILANHFPL SININNNFTL ENKHNNSIND ILLTDNTPGV SFYKNQLKAD **AAF13495**

YKIKLYCLCP NSLLANFIEL APNLNSNFII QEK-NNYTKD ILIPSSEAYG KFSKNINFKK **AAF34137**

YEIKLYCLCP NSLLANFIEL APNLNSNFII QEK-NNYTKD ILIPSSEAYG KFTKNINFKK **AAL06004**

YDVNIYSLNS D----EYFKL APDIGSDFVL SKKPKKYIND ILIPDKYAQE RYYG----KK **AAK73183**

YKIKLYCLCP NSLLANFIEL APNLNSNFII QEK-NNYTKD ILIPSSEAYG KFSKNINFKK **AAF31771**

NKIRIFKGR- ---------- ---------- ---------- ---------- ---------- **Q11201**

NKIRIFKGR- ---------- ---------- ---------- ---------- ---------- **P54751**

EKIKFFKGR- ---------- ---------- ---------- ---------- ---------- **Q11200**

NKIRIFKGR- ---------- ---------- ---------- ---------- ---------- **Q02745**

SKIEVYRGN- ---------- ---------- ---------- ---------- ---------- **Q16842**

SKIEVYRGN- ---------- ---------- ---------- ---------- ---------- **NP_835149**

GAIKNLTSF- ---------- ---------- ---------- ---------- ---------- **Q11203**

GAVKNLTYF- ---------- ---------- ---------- ---------- ---------- **P97325**

RVITDLSSGI ---------- ---------- ---------- ---------- ---------- **Q02734**

RVITDLSSGI ---------- ---------- ---------- ---------- ---------- **Q11206**

RVITDLSSGI ---------- ---------- ---------- ---------- ---------- **NP_033204**

GVVKDLSGGI DREF------ ---------- ---------- ---------- ---------- **Q9UNP4**

GVVEDLSGGI H--------- ---------- ---------- ---------- ---------- **O88829**

NLVINLTQD- ---------- ---------- ---------- ---------- ---------- **Q9Y274**

310 320 330 340 350 360

....|....| ....|....| ....|....| ....|....| ....|....| ....|....|

---------- ---------- ---------- ---------- ---------- ---------- **1RO7A**

NKIMLNFYNI LHSKDNLIKF LNKEIAVLKK QTTQRAKARI QNHLSYKLGQ ALIINSKSVL **AAF13495**

IKIKENVY-- ---------- ---------- ---------- ---------- ---------- **AAF34137**

IKIKENIY-- ---------- ---------- ---------- ---------- ---------- **AAL06004**

NKIMLNFYNI LHSKDNLIKF LNKEIAVLKK QTTQRAKARI QNHLSYKLGQ ALIINSKSVL **AAF13495**

IKIKENVY-- ---------- ---------- ---------- ---------- ---------- **AAF34137**

IKIKENIY-- ---------- ---------- ---------- ---------- ---------- **AAL06004**

SRLKENLH-- ---------- ---------- ---------- ---------- ---------- **AAK73183**

IKIKENIY-- ---------- ---------- ---------- ---------- ---------- **AAF31771**

---------- ---------- ---------- ---------- ---------- ---------- **Q11201**

---------- ---------- ---------- ---------- ---------- ---------- **P54751**

---------- ---------- ---------- ---------- ---------- ---------- **Q11200**

---------- ---------- ---------- ---------- ---------- ---------- **Q02745**

---------- ---------- ---------- ---------- ---------- ---------- **Q16842**

---------- ---------- ---------- ---------- ---------- ---------- **NP_835149**

---------- ---------- ---------- ---------- ---------- ---------- **Q11203**

---------- ---------- ---------- ---------- ---------- ---------- **P97325**

---------- ---------- ---------- ---------- ---------- ---------- **Q02734**

---------- ---------- ---------- ---------- ---------- ---------- **Q11206**

---------- ---------- ---------- ---------- ---------- ---------- **NP_033204**

---------- ---------- ---------- ---------- ---------- ---------- **Q9UNP4**

---------- ---------- ---------- ---------- ---------- ---------- **O88829**

---------- ---------- ---------- ---------- ---------- ---------- **Q9Y274**

370 380 390 400 410 420

....|....| ....|....| ....|....| ....|....| ....|....| ....|....|

---------- ---------- ---------- ---------- ---------- ---------- **1RO7A**

GFLSLPFIIL SIVISHKQEQ KAYKFKVKKN PNLALPPLET YPDYNEALKE KECFTYKLGE **AAF13495**

---------- ---------- ---------- ---------- ---------- -----YKLIK **AAF34137**

---------- ---------- ---------- ---------- ---------- -----YKLIK **AAL06004**

GFLSLPFIIL SIVISHKQEQ KAYKFKVKKN PNLALPPLET YPDYNEALKE KECFTYKLGE **AAF13495**

---------- ---------- ---------- ---------- ---------- -----YKLIK **AAF34137**

---------- ---------- ---------- ---------- ---------- -----YKLIK **AAL06004**

---------- ---------- ---------- ---------- ---------- -----YKLIK **AAK73183**

---------- ---------- ---------- ---------- ---------- -----YKLIK **AAF31771**

---------- ---------- ---------- ---------- ---------- ---------- **Q11201**

---------- ---------- ---------- ---------- ---------- ---------- **P54751**

---------- ---------- ---------- ---------- ---------- ---------- **Q11200**

---------- ---------- ---------- ---------- ---------- ---------- **Q02745**

---------- ---------- ---------- ---------- ---------- ---------- **Q16842**

---------- ---------- ---------- ---------- ---------- ---------- **NP_835149**

---------- ---------- ---------- ---------- ---------- ---------- **Q11203**

---------- ---------- ---------- ---------- ---------- ---------- **P97325**

---------- ---------- ---------- ---------- ---------- ---------- **Q02734**

---------- ---------- ---------- ---------- ---------- ---------- **Q11206**

---------- ---------- ---------- ---------- ---------- ---------- **NP_033204**

---------- ---------- ---------- ---------- ---------- ---------- **Q9UNP4**

---------- ---------- ---------- ---------- ---------- ---------- **O88829**

---------- ---------- ---------- ---------- ---------- ---------- **Q9Y274**

430 440 450

....|....| ....|....| ....|....| ....|....

---------- ---------- ---------- --------- **1RO7A**

EFIKAGKNWY GEGYIKFIFK DVPRLKREFE KGE------ **AAF13495**

DLLR------ ---------- -LPSDIKHYF KGK------ **AAF34137**

DLLK------ ---------- -LPSDIKHYF KGK------ **AAL06004**

EFIKAGKNWY GEGYIKFIFK DVPRLKREFE KGE------ **AAF13495**

DLLR------ ---------- -LPSDIKHYF KGK------ **AAF34137**

DLLK------ ---------- -LPSDIKHYF KGK------ **AAL06004**

DLIR------ ---------- -LPSDIKHYL KEKYANKNR **AAK73183**

DLLR------ ---------- -LPSDIKHYF KGK------ **AAF31771**

---------- ---------- ---------- --------- **Q11201**

---------- ---------- ---------- --------- **P54751**

---------- ---------- ---------- --------- **Q11200**

---------- ---------- ---------- --------- **Q02745**

---------- ---------- ---------- --------- **Q16842**

---------- ---------- ---------- --------- **NP_835149**

---------- ---------- ---------- --------- **Q11203**

---------- ---------- ---------- --------- **P97325**

---------- ---------- ---------- --------- **Q02734**

---------- ---------- ---------- --------- **Q11206**

---------- ---------- ---------- --------- **NP_033204**

---------- ---------- ---------- --------- **Q9UNP4**

---------- ---------- ---------- --------- **O88829**

---------- ---------- ---------- --------- **Q9Y274**
